# Three new species of *Ametadoria* Townsend (Diptera: Tachinidae) from Area de Conservación Guanacaste, Costa Rica

**DOI:** 10.3897/BDJ.3.e5039

**Published:** 2015-08-10

**Authors:** AJ Fleming, D. Monty Wood, M. Alex Smith, Winnie Hallwachs, Daniel Janzen

**Affiliations:** ‡ Canadian National Collection of Insects, Agriculture and Agri-Food Canada, Ottawa, Canada; § Department of Integrative Biology and the Biodiversity Institute of Ontario, Guelph, Canada; | University of Pennsylvania, Philadelphia, United States of America

**Keywords:** *
Ametadoria
*, *
Adidyma
*, *
Abolodoria
*, tropical rain forest, tropical dry forest, parasitoid fly, host-specificity, caterpillar

## Abstract

We describe three new species in the genus *Ametadoria* Townsend from Area de Conservación Guanacaste (ACG), Costa Rica. All three were reared from wild-caught Zygaenidae and Lacturidae caterpillars. We provide a concise description of each species using morphology, life history and molecular data, with photographic documentation. The new species are authored and described by Fleming and Wood: *Ametadoria karolramosae* **sp. nov.**, *Ametadoria leticiamartinezae* **sp. nov.**, and *Ametadoria mauriciogurdiani* **sp. nov.** The following are proposed by Wood as new synonyms of *Ametadoria* Townsend: *Adidyma* Townsend **syn. nov.**, and *Abolodoria* Townsend **syn. nov**. The following new combinations occur as a result of these new synonymies: *Ametadoria abdominalis* (Townsend) **comb. nov.**, *Ametadoria austrina* (Coquillett) **comb. nov.**, *Ametadoria humilis* (Wulp) **comb. nov.**, *Ametadoria misella* (Wulp) **comb. nov.** *Ametadoria adversa* (Townsend) is proposed as a junior synonym of ​*Ametadoria unispinosa* Townsend, **syn. nov**​.

## Introduction

The tachinid genus *Ametadoria* Townsend, 1927 is a small New World genus in the tribe Eryciini of the subfamily Exoristinae (Diptera: Tachinidae) (O'Hara and Wood 2004). The Eryciini are widely accepted as a polyphyletic “catch-all” assemblage of exoristine species with microtype eggs, which cannot be placed satisfactorily into other tribes (Crosskey 1967, Stireman 2002). To date there has been no definitive diagnosis of the tribe Eryciini, which is quite varied and very difficult to characterize. Stireman (2002) stated that there are no synapomorphies shared between the members of the tribe except for a trend towards ovo-larvipary. Crosskey (1967), however, provided some general traits that suggest the link between the genera included in the tribe: postpronotum with fewer than five bristles, katepimeron bare or at most with few sparse bristles, hind tibia irregularly bristled, vibrissa arising level with facial margin, inner margin of lower calypter curving away from scutellum, gena wider than profrons, antenna inserted well above middle of eye, and ocellar bristles never reclinate.

The genus *Ametadoria* was originally erected for a pair of female specimens collected in Itaquaquecetuba, Brasil, in 1927, by Townsend himself. The specific name *A. unispinosa* derives from the presence of a single spine at the base of wing-vein R_2+3_, a trait that occurs throughout the genus. *Ametadoria* is a specialist parasitoid of Lepidoptera caterpillars in the superfamily Zygaenoidea (Zygaenidae and Lacturidae). It ranges throughout the New World. Wood and Zumbado (2010) mentioned seven species in the genus *Ametadoria*, with only one known from the Nearctic (O'Hara and Wood 2004). This number of species comes from inclusion of several synonymies that were only speculative at the time of Wood and Zumbado (2010), but which are here officially proposed.

This work builds on existing knowledge and describes three new species of *Ametadoria* Townsend, all reared from wild-caught caterpillars from Area de Conservacíon Guanacaste (ACG), Costa Rica. It is part of a series of papers describing reared specimens from the ongoing inventory being conducted in ACG (Janzen et al. 2009, Fleming et al. 2014a, Fleming et al. 2014b, Fleming et al. 2015). The new species described here are based on differences in external morphology, COI (cox1 or cytochrome oxidase 1) gene sequences or “DNA barcodes”, and male terminalia. By using COI data in combination with morphological descriptions, we are able to show that markings on the abdomen are not just different between males and females but are also consistent within species, thereby making them an ideal tool for species differentiation. Based on examinations conducted by Wood, we also propose two new generic synonymies, resulting in five new combinations. We build on the existing knowledge base of *Ametadoria* by providing new records relating to its distribution and confirming the host preference of the genus.

## Materials and methods

### Acronyms for depositories

AMNH - American Museum of Natural History, New York, N.Y., USA

BMNH - The Natural History Museum, London, UK

CNC - Canadian National Collection of Insects, Arachnids and Nematodes, Ottawa, Canada

USNM - National Museum of Natural History, Washington, D.C., USA

MZSP - Museu de Zoologia Universidade de São Paulo, São Paulo, Brazil

INBio - Instituto Nacional de Biodiversidad, Santo Domingo de Heredia, Costa Rica

### Study area and rearing intensity

All flies and rearing information described here were found in the framework of the 35+ year–old ongoing inventory of the caterpillars, their food plants and their parasitoids, present in the various biomes (dry forest, rain forest, cloud forest and intergrades) of the 125,000+ ha terrestrial portion of Area de Conservación Guanacaste (ACG) in north western Costa Rica (Smith et al. 2005, Smith et al. 2006, Smith et al. 2007, Smith et al. 2008, Smith et al. 2009, Janzen et al. 2009, Janzen and Hallwachs 2011, Rodriguez et al. 2012, Smith et al. 2012). The parasitoid rearing methods are illustrated in brief at http://janzen.bio.upenn.edu/caterpillars/methodology/how/parasitoid_husbandry.htm. This inventory has reared more than 600,000 wild-caught caterpillars since 1978. All frequencies of parasitization reported here need to be considered against this background inventory (Janzen et al. 2009, Janzen and Hallwachs 2011, Fernandez-Triana et al. 2014).

### Imaging and dissections

Descriptions of new species discussed in this paper are deliberately brief, only including some basic descriptions of body parts and colors commonly used in tachinid fly identification. These brief descriptions are complemented with an extensive series of color photos of each species to illustrate the readily observed inter-specific differences.

Habitus and genitalia photographs were taken as outlined in Fleming et al. (2014a). The series of raw image files were first processed with Adobe Photoshop CS6, adjusting for white balance and contrast. This series was then digitally stacked using Zerene Stacker v.1.04, thereby maximizing image quality and depth of field, producing a final composite image.

Adult fly dissections followed standard practice (O’Hara 1983). Photographs of male terminalia were taken using a Canon S110 digital camera adaptor mounted to the eyepiece of a Leitz–Wetzlar dissecting microscope. Preparations were mounted on a depression slide in a small quantity of Rexall hand sanitizer gel (NPN# 80007138) (Fleming et al. 2014a). After mounting and photographing, the terminalia were rinsed in a small quantity of pure distilled water before being replaced in a glycerine-filled microvial attached to the pin.

The terminology used for components of the terminalia (which refers only to the sclerotized parts of the genitalia, and not to the soft internal structures) and other body parts follows Cumming and Wood (2009).

### Voucher specimen management

All caterpillars reared from the ACG efforts receive a unique voucher code in the format yy–SRNP–xxxxx. Any parasitoid emerging from this caterpillar receives the same voucher code. If and when it is later dealt with individually, it receives a second voucher code unique to it, in the format DHJPARxxxxxxx. The voucher codes assigned to both host as well as emerged parasitoids may be found at http://janzen.bio.upenn.edu/caterpillars/database.lasso. All DHJPARxxxxxxx coded tachinids have had one leg removed for attempted DNA barcoding at the Biodiversity Institute of Ontario (BIO) in Guelph, with all collateral data and all successful barcodes permanently deposited in the Barcode of Life Data System (BOLD, www.boldsystems.org) (Ratnasingham and Hebert 2007), and later migrated to GenBank as well. As the inventory is continually growing, new specimens can be found by searching the genus *Ametadoria* in BOLD. Each barcoded specimen has an accession code from the Barcode of Life Data System (BOLD) and GenBank.

Inventoried Tachinidae were collected under Costa Rican government research permits issued to DHJ, and likewise exported under permit by DHJ from Costa Rica to Philadelphia, and then to their final depository in the Canadian National Insect collection (CNC) in Ottawa, Canada. Tachinid identifications for the inventory were done by DHJ in coordination with a) visual inspection by AJF and DMW, b) DNA barcoding by MAS, as recorded in BIO and BOLD, and c) correlation with host caterpillar identifications by DHJ and WH through the inventory itself. Dates of capture of each specimen are the dates of eclosion of the fly, and not the date of capture of the caterpillar, since the fly eclosion date is much more representative of the time when that fly species is on the wing than is the time of capture of the caterpillar. However, the collector listed is the parataxonomist who found the caterpillar, rather than the person who retrieved the newly eclosed fly and processed it by freezing, pinning, labeling and oven-drying. The biology and natural history of these flies will be the subject of later papers.

### DNA barcoding

**Figure 1.**
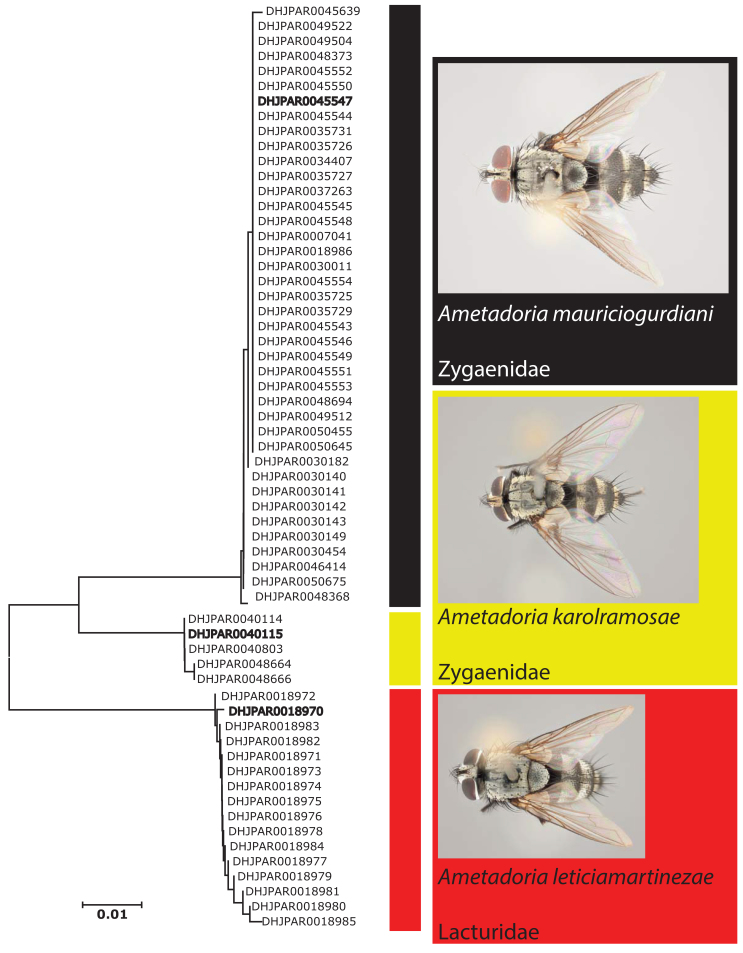
Neighbor-Joining (NJ – Saitou and Nei 1987) tree based on Kimura 2-parameter distances (K2P – Kimura 1980) made using MEGA6 (Tamura et al. 2013) for 62 specimens from three species of *Ametadoria* in ACG. Tip labels include specimen accession, the dorsal image of a male color-coded to its corresponding clade, and the host family. Holotype voucher codes appear in boldface.

DNA barcodes (the standardised 5’ region of the mitochondrial cytochrome c oxidase I (COI) gene) for all ACG inventory specimens were obtained using DNA extracts prepared from single legs using a glass fibre protocol (Ivanova et al. 2006). The DNA barcode region (a 658-bp region near the 5’ terminus of the COI gene) was amplified from extracted DNA using standard primers (LepF1–LepR1 (Penton et al. 2004)) and following established protocols (Smith et al. 2006, Smith et al. 2007, Smith et al. 2008). All information for the sequences associated with each individual specimen can be retrieved from the Barcode of Life Data System (BOLD) (Ratnasingham and Hebert 2007) by Process ID (sequence accession) or here: http://dx.doi.org/10.5883/DS-ASAMETA. A neighbor–joining (NJ) tree (Saitou and Nei 1987) for all *Ametadoria* specimens reared and DNA-barcoded to date by this inventory is shown in Fig. 1​.

### Specimens examined

In the process of species determination, specimens provided from the ACG were examined in comparison to the known New World species of *Ametadoria*, by both AJF and DMW. Differential characters are discussed in the diagnoses, when necessary. Where possible, holotypes of previously described species of *Ametadoria* were compared to ACG specimens; however, if a holotype specimen was unavailable for examination, this is noted in the description.

### Generic synonyms of *Ametadoria*

*Ametadoria* Townsend 1927: 276. Type species: *Ametadoria unispinosa* Townsend 1927, by original designation.

*Adidyma* Townsend 1935: 230. Type species: *Adidyma adversa* Townsend 1935, by original designation. **N. syn.**

*Abolodoria* Townsend 1934: 400. Type species: *Abolodoria abdominalis* Townsend 1934, by original designation. **N. syn.**

### Species included in *Ametadoria*

***abdominalis*** Townsend 1934: 400 (*Abolodoria*). Holotype female (USNM) [examined by DMW]. Type locality: Brazil, São Paulo, Itaquaquecetuba. **N. comb.**

***austrina*** Coquillett 1902: 113 (*Sturmia*). Holotype male (USNM) [examined by DMW]. Type locality: Bahamas, Nassau. **N. comb.**

***fuliginipennis*** Wulp 1890a: 164 (*Didyma*). Holotype male (BMNH) [examined by DMW]. Type locality: Mexico, Guerrero, Omilteme, 8000ft. Combination established by Wood 1985).

***harrisinae*** Coquillett 1897: 111 (*Sturmia*). Holotype female (US NM) [examined by DM W]. Type locality: United States. Synonymy established by O’Hara and Wood 1998).

*tuxedo* Curran 1930: 102 (*Erycia*). Holotype male (AMNH) [examined by DMW]. Type locality: USA, New York, Tuxedo, Station for the Study of Insects. Included in Sabrosky and Arnaud 1965) as *Sturmia*, but not marked as a new combination.

*unispinosa* Reinhard 1930: 199 (*Masicera*). Holotype male (USNM) [examined by DMW]. Type locality: USA, Texas, Co llege Station. Included in Sabrosky and Arnaud 1965) as *Sturmia*​, but not mark ed as a new combination.

***humilis*** Wulp 1890c: 72 (*Exorista*). Holotype male (BMNH) [examined by DMW]. Type locality: Mexico, Guerrero, Omilteme, 8000ft. **N. comb.**

***misella*** Wulp 1890b: 204 (*Anisia*). 2 female syntypes (BMNH) [examined by DMW]. Type locality: Mexico, Guerrero, Amula, 6000ft. **N. comb.** This name has been erroneously listed in the literature as a synonym of *A. harrisinae*. *Ametadoria misella* can be distinguished from *A. harrisinae* by the presence of bristles extending halfway up the facial ridge, and having a much wider fronto-orbital plate in males relative to *A. harrisinae*.

***unispinosa*** Townsend 1927: 285 (*Ametadoria*). 2 female syntypes (USNM) [examined by DMW]. Localities: Brazil, São Paulo, Itaquaquecetuba.

*adversa* Townsend 1935: 231 (*Adidyma*). Holotype female (MZSP) [examined by DMW]. Type locality: Brazil, São Paulo, São Vicente. **N. syn.**

### Diagnosis of the genus *Ametadoria*

The tachinid genus *Ametadoria* Townsend was originally based on two female syntypes. *Ametadoria* is a specialist parasitoid of Lepidoptera caterpillars in Zygaenoidea (Zygaenidae and Lacturidae). Adapting Townsend’s original diagnosis of *Ametadoria*, combined with current observations, the genus can be recognized by the following traits (differences between the sexes are noted where applicable). **Head:** eyes bare; ocellar bristles well developed and proclinate, arising behind anterior ocellus; anterior half of palpus yellow-orange, darkening to gray-black basally; palpus can be entirely covered in setulae or partially bare; fronto-orbital plate silver to very slightly gold tinged; fronto-orbital plate bare or with fine hairs interspersed with bristles; fronto-orbital plate (measured at the height of the scape) between 1.5–4X as wide as frontal vitta; parafacial and gena silvery to slightly brassy in color; gena 1/8^th^ to 1/12^th^ height of head; males with 2–3 reclinate upper orbital bristles, the anteriormost bristle being the largest; females with 2 proclinate orbital bristles; frontal row of bristles extending up to or slightly beyond base of arista; vibrissa arising at facial margin or slightly above it, with 4–5 supravibrissal bristles along the facial ridge; antenna dark-brown to black; arista minutely pubescent to bare at base and bare apically, concolorous with 1^st ^flagellomere. **Thorax:** prosternum setulose; thorax and scutellum silvery gray throughout with 4 prominent thoracic vittae; 3–4 post-pronotal bristles; 4 post-sutural dorsocentrals; 3 post-sutural supra-alars; meron bearing 6 or more tightly packed bristles sometimes interspersed with finer hairs; katepimeron bare; 3 katepisternal bristles; 1 pair of discal bristles on scutellum; apical scutellars at least 1/2 the length of subapicals and crossed at their apex; subapical scutellars parallel to very slightly convergent. Wing: smoky yellow to pale with all veins bare except for one setula at the base of R_2+3_; calypters translucent white to smoky yellow; legs entirely black. **Abdomen:** with gold or silver tomentum on anterior 1/2 of tergites 3, 4 and 5; mid-dorsal depression extending to the posterior margin of syntergite 1+2; 1 pair of median marginal bristles on tg 3 and a row of marginals on tg 4 and tg 5; no abdominal discals; sex patches of densely adpressed short hairs present on tg 4 and tg 5 (see Cerretti et al. 2015). In females, abdominal tergites dark velvety black, bright yellowish bands covering up to 1/2 or more of tergal surface along anterior margin of tergites 3, 4 and 5. **Male terminalia:** sternite 5 with deep median cleft forming two rounded outer lobes, these covered in dense tomentum marginally; lobes of sternite 5 bearing several long bristles. Cerci appearing dorsally flattened, and narrow in posterior view; cerci not fused medially, with an almost equidistant separation along their entire length. Surstylus short and truncate, 1/2 to 2/3 as long as cerci; surstylus appearing rounded and sub-triangular with a slight hook to the tip when viewed laterally, tip sometimes slightly haired on its margin; posterior half of lateral surface of surstylus with several stout bristles. Phallus short and stout, and subequal to length of cerci. Postgonite from 1/3 to 1/2 as long as phalloapodeme.

*Ametadoria* Townsend is distinguished from its sister taxa *Lydella* Robineau-Desvoidy and *Drino* Robineau-Desvoidy by its bare eyes, the presence of three katepisternal bristles, a single row of frontal bristles, and the anteriormost reclinate orbital bristle larger than posterior reclinate orbitals. Males and females within this genus are slightly dimorphic, with females possessing 2 proclinate fronto-orbital bristles, a wider fronto-orbital plate, and striking differences in coloration as well as pattern.

## Taxon treatments

### Ametadoria karolramosae

Fleming & Wood sp. n.

urn:lsid:zoobank.org:act:023E0520-B1AC-4D15-B528-C9B2B0E372F4

#### Materials

- **Type status:** Holotype. **Occurrence:** occurrenceDetails: http://janzen.sas.upenn.edu; catalogNumber: DHJPAR0040115; recordedBy: D.H. Janzen, W. Hallwachs & Guillermo Pereira; individualID: DHJPAR0040115; individualCount: 1; sex: M; lifeStage: adult; preparations: pinned; otherCatalogNumbers: 10-SRNP-13805; **Taxon:** scientificName: Ametadoria karolramosae; phylum: Arthropoda; class: Insecta; order: Diptera; family: Tachinidae; genus: Ametadoria; specificEpithet: karolramosae; scientificNameAuthorship: Fleming & Wood, 2015; **Location:** continent: Central America; country: Costa Rica; countryCode: CR; stateProvince: Guanacaste; county: Area de Conservación Guanacaste; locality: Sector Santa Rosa; verbatimLocality: Area Administrativa; verbatimElevation: 295; verbatimLatitude: 10.838; verbatimLongitude: -85.619; verbatimCoordinateSystem: Decimal; decimalLatitude: 10.838; decimalLongitude: -85.619; **Identification:** identifiedBy: Fleming & Wood; dateIdentified: 2014; **Event:** samplingProtocol: reared from caterpillar of unidentified Zygaenidae; verbatimEventDate: Aug-24-2010; **Record Level:** language: en; institutionCode: CNC; collectionCode: Insects; basisOfRecord: Pinned Specimen
- **Type status:** Paratype. **Occurrence:** occurrenceDetails: http://janzen.sas.upenn.edu; catalogNumber: DHJPAR0040803; recordedBy: D.H. Janzen, W. Hallwachs & Guillermo Pereira; individualID: DHJPAR0040803; individualCount: 1; sex: M; lifeStage: adult; preparations: pinned; otherCatalogNumbers: 10-SRNP-13780; **Taxon:** scientificName: Ametadoria karolramosae; phylum: Arthropoda; class: Insecta; order: Diptera; family: Tachinidae; genus: Ametadoria; specificEpithet: karolramosae; scientificNameAuthorship: Fleming & Wood, 2015; **Location:** continent: Central America; country: Costa Rica; countryCode: CR; stateProvince: Guanacaste; county: Area de Conservación Guanacaste; locality: Sector Santa Rosa; verbatimLocality: Area Administrativa; verbatimElevation: 295; verbatimLatitude: 10.838; verbatimLongitude: -85.619; verbatimCoordinateSystem: Decimal; decimalLatitude: 10.838; decimalLongitude: -85.619; **Identification:** identifiedBy: Fleming & Wood; dateIdentified: 2014; **Event:** samplingProtocol: reared from caterpillar of unidentified Zygaenidae; verbatimEventDate: Sep-06-2010; **Record Level:** language: en; institutionCode: CNC; collectionCode: Insects; basisOfRecord: Pinned Specimen
- **Type status:** Paratype. **Occurrence:** occurrenceDetails: http://janzen.sas.upenn.edu; catalogNumber: DHJPAR0040114; recordedBy: D.H. Janzen, W. Hallwachs & Guillermo Pereira; individualID: DHJPAR0040114; individualCount: 1; sex: F; lifeStage: adult; preparations: pinned; otherCatalogNumbers: 10-SRNP-13800; **Taxon:** scientificName: Ametadoria karolramosae; phylum: Arthropoda; class: Insecta; order: Diptera; family: Tachinidae; genus: Ametadoria; specificEpithet: karolramosae; scientificNameAuthorship: Fleming & Wood, 2015; **Location:** continent: Central America; country: Costa Rica; countryCode: CR; stateProvince: Guanacaste; county: Area de Conservación Guanacaste; locality: Sector Santa Rosa; verbatimLocality: Area Administrativa; verbatimElevation: 295; verbatimLatitude: 10.838; verbatimLongitude: -85.619; verbatimCoordinateSystem: Decimal; decimalLatitude: 10.838; decimalLongitude: -85.619; **Identification:** identifiedBy: Fleming & Wood; dateIdentified: 2014; **Event:** samplingProtocol: reared from caterpillar of unidentified Zygaenidae; verbatimEventDate: Aug-24-2010; **Record Level:** language: en; institutionCode: CNC; collectionCode: Insects; basisOfRecord: Pinned Specimen

#### Description

**Male** (Fig. 2a, b, c). **Head** (Fig. 2c): frontal vitta dark black, narrowed apically to slightly less than the width of the ocellar triangle, fronto-orbital plate 2X as wide as frontal vitta; ocellar bristles half as long as arista; frontal bristles extending below level of arista by width of one bristle; small black hairs intermingled with frontal bristles all along the parafrontal, extending to level of pedicel; antenna black; arista black, minutely pubescent at its base, and bare at its tip; proclinate orbital bristles absent; fronto-orbital plate mostly silver, except for gold tinge along the margin of the frontal vitta; parafacial silver; palpus yellow and haired at its tip; gena 1/10 height of head. **Thorax** (Fig. 2a, b): grayish-gold when viewed dorsally, with four longitudinal dark gray vittae; scutellum bearing slightly yellow gray tomentosity occupying 1/2 or more of its distal end, darkened along its anterior edge (this tomentosity extending to underside of scutellum). Legs black; anteroventral surface of hind femur bearing 5–6 long erect bristles of irregular length and irregularly spaced; anteroventral surface of hind tibia with tightly adpressed, short and regularly spaced hairs, anterodorsal surface with a regularly spaced comb of bristles 2X as long as leg is wide, 1 bristle on each of the anterodorsal, anteroventral, posterodorsal and posteroventral surfaces of the tibia, 2X as long as the surrounding bristles. Wing (Fig. 2a): pale smoky yellowish in color, with 1 solitary spine at the base of R_2+__3_; calypter pale translucent, haired along its margins. **Abdomen** (Fig. 2a): ground color of abdominal tergites dark brown, appearing shiny black dorsally, with bright brassy tomentose bands covering up to 1/2 of the anterior margins of abdominal tg 3, 4, and 5, these bands wrapping around to the underside of the abdomen; tg 3, 4 and 5 possessing median marginal bristles, syntergite 1+2 possessing reduced median marginals, only 2X as long as abdominal setulae. Ventrolateral surface of syntergite 1+2, tg 3, and tg 4 orange. Sex patches present on ventral surface of tg 4, and tg 5. **Terminalia** (Figs 5a, 6a, 7a): sternite 5 with deep U-shaped median cleft, forming two rounded outer lobes, these covered in dense tomentum; lobes of sternite 5 bearing 3 long bristles marginally, approximately 3X as long as other bristles present on sternite 5. Cerci almost flat when viewed laterally, narrow and finger-like in posterior view, not fused medially, maintaining an almost equidistant separation along their entire length; cerci covered in long setulae dorsally, tapering at around 2/3 the length of the appendage, and with apex bare. Surstylus short and truncate, approximately 1/2 as long as cerci, appearing rounded and sub-triangular with a slight hook to the tip when viewed laterally; distal half of lateral surface of surstylus with several stout bristles. Epandrium when viewed laterally extending backwards, 3/4 length of cerci. Phallus short and stout, approximately the same length as cerci. Postgonite 1/2 as long as phalloapodeme with a moderate forward curve.

**Female** (Fig. 2d, e, f). **Head** (Fig. 2f): frontal vitta dark black, parallel-sided, less than the width of the ocellar triangle; fronto-orbital plate 3X as wide as frontal vitta; frontal bristles not reaching below level of arista; black setulae on fronto-orbital plate extending to level of pedicel; 2 proclinate orbital bristles present; arista black and bare; parafrontal with golden tomentum covering over 80% of its surface; parafacial silvery; palpi yellow and haired. **Thorax** (Fig. 2d, e): grayish, with distinctive brassy tomentosity apparent when viewed under the microscope; four longitudinal black vittae, the middle pair extending only down 1/3 of thorax, postsuturally; scutellum bearing white or yellowish tomentosity over 4/5 of its surface. Legs and wings as in males. **Abdomen** (Fig. 2d​): abdominal tergites dark velvety black, with bright yellowish bands covering up to 1/2 or more of tergal surface along anterior margin of tergites 3, 4 and 5; syntergite 1+2, and tergites 3, 4 and 5 possessing median marginal bristles, reduced on syntergite 1+2.

#### Diagnosis

Fronto-orbital plate with golden tomentum along the margin of the frontal vitta, parafacial silver. Abdominal tergites 3, 4, and 5 bearing a brassy tomentosity on anterior margin, extending at least along 1/2 of tergal surface, with females bearing additional brassy tomentosity on thorax. Calypters pale translucent. Hind tibia with a regularly spaced comb of hairs 2X as long as tibia is wide. Ventrolateral surface of syntergite 1+2, 3 and 4 with orange ground color. Syntergite 1+2 possessing reduced median marginal bristles, only 2X as long as abdominal hairs. Epandrium when viewed laterally extending backwards, 3/4 length of cerci; phallus short and stout, approximately same length as cerci; postgonite 1/2 as long as phalloapodeme with a moderate forward curve. *Ametadoria karolramosae* is differentiated from its most similar congener, *A. unispinosa*, by the following traits: the presence of marginal bristles on syntergite 1+2, the ventrolateral orange ground color on the abdomen, and its pale translucent calypters.

#### Etymology

*Ametadoria karolramosae* is named in recognition of Karol Ramos Méndez for her contributions to the accounting team for Area de Conservación Guanacaste, the forest this fly lives in.

#### Distribution

Costa Rica, ACG, Prov. Guanacaste, dry forest, 295m elevation.

#### Ecology

Host: *Ametadoria karolramosae* was reared from one species of unidentified Zygaenidae feeding on *Cissus alata* (Vitaceae).

### Ametadoria leticiamartinezae

Fleming & Wood sp. n.

urn:lsid:zoobank.org:act:66AEEC7F-8BA2-42B9-B8C3-F8F924C0CB48

#### Materials

- **Type status:** Holotype. **Occurrence:** occurrenceDetails: http://janzen.sas.upenn.edu; catalogNumber: DHJPAR0018970; recordedBy: D.H. Janzen, W. Hallwachs & gusaneros; individualID: DHJPAR0018970; individualCount: 1; sex: M; lifeStage: adult; preparations: pinned; otherCatalogNumbers: 02-SRNP-11285; **Taxon:** scientificName: Ametadoria leticiamartinezae; phylum: Arthropoda; class: Insecta; order: Diptera; family: Tachinidae; genus: Ametadoria; specificEpithet: leticiamartinezae; scientificNameAuthorship: Fleming & Wood, 2015; **Location:** continent: Central America; country: Costa Rica; countryCode: CR; stateProvince: Guanacaste; county: Area de Conservación Guanacaste; locality: Sector Santa Elena; verbatimLocality: Vado Quebrada Calera; verbatimElevation: 305; verbatimLatitude: 10.867; verbatimLongitude: -85.647; verbatimCoordinateSystem: Decimal; decimalLatitude: 10.867; decimalLongitude: -85.647; **Identification:** identifiedBy: Fleming & Wood; dateIdentified: 2014; **Event:** samplingProtocol: reared from caterpillar of Lactura subfervensDHJ01 (Lacturidae); verbatimEventDate: 02-Sep-2002; **Record Level:** language: en; institutionCode: CNC; collectionCode: Insects; basisOfRecord: Pinned Specimen
- **Type status:** Paratype. **Occurrence:** occurrenceDetails: http://janzen.sas.upenn.edu; catalogNumber: DHJPAR0018981; recordedBy: D.H. Janzen, W. Hallwachs & gusaneros; individualID: DHJPAR0018981; individualCount: 1; sex: F; lifeStage: adult; preparations: pinned; otherCatalogNumbers: 96-SRNP-296; **Taxon:** scientificName: Ametadoria leticiamartinezae; phylum: Arthropoda; class: Insecta; order: Diptera; family: Tachinidae; genus: Ametadoria; specificEpithet: leticiamartinezae; scientificNameAuthorship: Fleming & Wood, 2015; **Location:** continent: Central America; country: Costa Rica; countryCode: CR; stateProvince: Guanacaste; county: Area de Conservación Guanacaste; locality: Sector Murcielago; verbatimLocality: Pozo Del General; verbatimElevation: 40; verbatimLatitude: 10.898; verbatimLongitude: -85.731; verbatimCoordinateSystem: Decimal; decimalLatitude: 10.898; decimalLongitude: -85.731; **Identification:** identifiedBy: Fleming & Wood; dateIdentified: 2014; **Event:** samplingProtocol: reared from caterpillar of Lactura subfervensDHJ01 (Lacturidae); verbatimEventDate: 01-Jun-1996; **Record Level:** language: en; institutionCode: CNC; collectionCode: Insects; basisOfRecord: Pinned Specimen
- **Type status:** Paratype. **Occurrence:** occurrenceDetails: http://janzen.sas.upenn.edu; catalogNumber: DHJPAR0018973; recordedBy: D.H. Janzen, W. Hallwachs & gusaneros; individualID: DHJPAR0018973; individualCount: 1; sex: F; lifeStage: adult; preparations: pinned; otherCatalogNumbers: 02-SRNP-11281; **Taxon:** scientificName: Ametadoria leticiamartinezae; phylum: Arthropoda; class: Insecta; order: Diptera; family: Tachinidae; genus: Ametadoria; specificEpithet: leticiamartinezae; scientificNameAuthorship: Fleming & Wood, 2015; **Location:** continent: Central America; country: Costa Rica; countryCode: CR; stateProvince: Guanacaste; county: Area de Conservación Guanacaste; locality: Sector Santa Elena; verbatimLocality: Vado Quebrada Calera; verbatimElevation: 305; verbatimLatitude: 10.867; verbatimLongitude: -85.647; verbatimCoordinateSystem: Decimal; decimalLatitude: 10.867; decimalLongitude: -85.647; **Identification:** identifiedBy: Fleming & Wood; dateIdentified: 2014; **Event:** samplingProtocol: reared from caterpillar of Lactura subfervensDHJ01 (Lacturidae); verbatimEventDate: 16-Aug-2002; **Record Level:** language: en; institutionCode: CNC; collectionCode: Insects; basisOfRecord: Pinned Specimen
- **Type status:** Paratype. **Occurrence:** occurrenceDetails: http://janzen.sas.upenn.edu; catalogNumber: DHJPAR0018978; recordedBy: D.H. Janzen, W. Hallwachs & gusaneros; individualID: DHJPAR0018978; individualCount: 1; sex: M; lifeStage: adult; preparations: pinned; otherCatalogNumbers: 02-SRNP-11286; **Taxon:** scientificName: Ametadoria leticiamartinezae; phylum: Arthropoda; class: Insecta; order: Diptera; family: Tachinidae; genus: Ametadoria; specificEpithet: leticiamartinezae; scientificNameAuthorship: Fleming & Wood, 2015; **Location:** continent: Central America; country: Costa Rica; countryCode: CR; stateProvince: Guanacaste; county: Area de Conservación Guanacaste; locality: Sector Santa Elena; verbatimLocality: Vado Quebrada Calera; verbatimElevation: 305; verbatimLatitude: 10.867; verbatimLongitude: -85.647; verbatimCoordinateSystem: Decimal; decimalLatitude: 10.867; decimalLongitude: -85.647; **Identification:** identifiedBy: Fleming & Wood; dateIdentified: 2014; **Event:** samplingProtocol: reared from caterpillar of Lactura subfervensDHJ01 (Lacturidae); verbatimEventDate: 24-Aug-2002; **Record Level:** language: en; institutionCode: CNC; collectionCode: Insects; basisOfRecord: Pinned Specimen
- **Type status:** Paratype. **Occurrence:** occurrenceDetails: http://janzen.sas.upenn.edu; catalogNumber: DHJPAR0018979; recordedBy: D.H. Janzen, W. Hallwachs & gusaneros; individualID: DHJPAR0018979; individualCount: 1; sex: M; lifeStage: adult; preparations: pinned; otherCatalogNumbers: 96-SRNP-322; **Taxon:** scientificName: Ametadoria leticiamartinezae; phylum: Arthropoda; class: Insecta; order: Diptera; family: Tachinidae; genus: Ametadoria; specificEpithet: leticiamartinezae; scientificNameAuthorship: Fleming & Wood, 2015; **Location:** continent: Central America; country: Costa Rica; countryCode: CR; stateProvince: Guanacaste; county: Area de Conservación Guanacaste; locality: Sector Murcielago; verbatimLocality: Pozo Del General; verbatimElevation: 40; verbatimLatitude: 10.898; verbatimLongitude: -85.731; verbatimCoordinateSystem: Decimal; decimalLatitude: 10.898; decimalLongitude: -85.731; **Identification:** identifiedBy: Fleming & Wood; dateIdentified: 2014; **Event:** samplingProtocol: reared from caterpillar of Lactura subfervensDHJ01 (Lacturidae); verbatimEventDate: 15-Jun-1996; **Record Level:** language: en; institutionCode: CNC; collectionCode: Insects; basisOfRecord: Pinned Specimen
- **Type status:** Paratype. **Occurrence:** occurrenceDetails: http://janzen.sas.upenn.edu; catalogNumber: DHJPAR0018980; recordedBy: D.H. Janzen, W. Hallwachs & gusaneros; individualID: DHJPAR0018980; individualCount: 1; sex: M; lifeStage: adult; preparations: pinned; otherCatalogNumbers: 96-SRNP-300; **Taxon:** scientificName: Ametadoria leticiamartinezae; phylum: Arthropoda; class: Insecta; order: Diptera; family: Tachinidae; genus: Ametadoria; specificEpithet: leticiamartinezae; scientificNameAuthorship: Fleming & Wood, 2015; **Location:** continent: Central America; country: Costa Rica; countryCode: CR; stateProvince: Guanacaste; county: Area de Conservación Guanacaste; locality: Sector Murcielago; verbatimLocality: Pozo Del General; verbatimElevation: 40; verbatimLatitude: 10.898; verbatimLongitude: -85.731; verbatimCoordinateSystem: Decimal; decimalLatitude: 10.898; decimalLongitude: -85.731; **Identification:** identifiedBy: Fleming & Wood; dateIdentified: 2014; **Event:** samplingProtocol: reared from caterpillar of Lactura subfervensDHJ01 (Lacturidae); verbatimEventDate: 05-Jun-1996; **Record Level:** language: en; institutionCode: CNC; collectionCode: Insects; basisOfRecord: Pinned Specimen
- **Type status:** Paratype. **Occurrence:** occurrenceDetails: http://janzen.sas.upenn.edu; catalogNumber: DHJPAR0018971; recordedBy: D.H. Janzen, W. Hallwachs & gusaneros; individualID: DHJPAR0018971; individualCount: 1; sex: M; lifeStage: adult; preparations: pinned; otherCatalogNumbers: 02-SRNP-10747; **Taxon:** scientificName: Ametadoria leticiamartinezae; phylum: Arthropoda; class: Insecta; order: Diptera; family: Tachinidae; genus: Ametadoria; specificEpithet: leticiamartinezae; scientificNameAuthorship: Fleming & Wood, 2015; **Location:** continent: Central America; country: Costa Rica; countryCode: CR; stateProvince: Guanacaste; county: Area de Conservación Guanacaste; locality: Sector Santa Elena; verbatimLocality: Vado Quebrada Calera; verbatimElevation: 305; verbatimLatitude: 10.867; verbatimLongitude: -85.647; verbatimCoordinateSystem: Decimal; decimalLatitude: 10.867; decimalLongitude: -85.647; **Identification:** identifiedBy: Fleming & Wood; dateIdentified: 2014; **Event:** samplingProtocol: reared from caterpillar of Lactura subfervensDHJ01 (Lacturidae); verbatimEventDate: 14-Oct-2002; **Record Level:** language: en; institutionCode: CNC; collectionCode: Insects; basisOfRecord: Pinned Specimen
- **Type status:** Paratype. **Occurrence:** occurrenceDetails: http://janzen.sas.upenn.edu; catalogNumber: DHJPAR0018974; recordedBy: D.H. Janzen, W. Hallwachs & gusaneros; individualID: DHJPAR0018974; individualCount: 1; sex: F; lifeStage: adult; preparations: pinned; otherCatalogNumbers: 02-SRNP-11311; **Taxon:** scientificName: Ametadoria leticiamartinezae; phylum: Arthropoda; class: Insecta; order: Diptera; family: Tachinidae; genus: Ametadoria; specificEpithet: leticiamartinezae; scientificNameAuthorship: Fleming & Wood, 2015; **Location:** continent: Central America; country: Costa Rica; countryCode: CR; stateProvince: Guanacaste; county: Area de Conservación Guanacaste; locality: Sector Santa Elena; verbatimLocality: Vado Quebrada Calera; verbatimElevation: 305; verbatimLatitude: 10.867; verbatimLongitude: -85.647; verbatimCoordinateSystem: Decimal; decimalLatitude: 10.867; decimalLongitude: -85.647; **Identification:** identifiedBy: Fleming & Wood; dateIdentified: 2014; **Event:** samplingProtocol: reared from caterpillar of Lactura subfervensDHJ01 (Lacturidae); verbatimEventDate: 18-Aug-2002; **Record Level:** language: en; institutionCode: CNC; collectionCode: Insects; basisOfRecord: Pinned Specimen
- **Type status:** Paratype. **Occurrence:** occurrenceDetails: http://janzen.sas.upenn.edu; catalogNumber: DHJPAR0018982; recordedBy: D.H. Janzen, W. Hallwachs & gusaneros; individualID: DHJPAR0018982; individualCount: 1; sex: F; lifeStage: adult; preparations: pinned; otherCatalogNumbers: 96-SRNP-164; **Taxon:** scientificName: Ametadoria leticiamartinezae; phylum: Arthropoda; class: Insecta; order: Diptera; family: Tachinidae; genus: Ametadoria; specificEpithet: leticiamartinezae; scientificNameAuthorship: Fleming & Wood, 2015; **Location:** continent: Central America; country: Costa Rica; countryCode: CR; stateProvince: Guanacaste; county: Area de Conservación Guanacaste; locality: Sector Murcielago; verbatimLocality: Pozo Del General; verbatimElevation: 40; verbatimLatitude: 10.898; verbatimLongitude: -85.731; verbatimCoordinateSystem: Decimal; decimalLatitude: 10.898; decimalLongitude: -85.731; **Identification:** identifiedBy: Fleming & Wood; dateIdentified: 2014; **Event:** samplingProtocol: reared from caterpillar of Lactura subfervensDHJ01 (Lacturidae); verbatimEventDate: 24-Feb-1996; **Record Level:** language: en; institutionCode: CNC; collectionCode: Insects; basisOfRecord: Pinned Specimen
- **Type status:** Paratype. **Occurrence:** occurrenceDetails: http://janzen.sas.upenn.edu; catalogNumber: DHJPAR0018977; recordedBy: D.H. Janzen, W. Hallwachs & gusaneros; individualID: DHJPAR0018977; individualCount: 1; sex: F; lifeStage: adult; preparations: pinned; otherCatalogNumbers: 02-SRNP-11297; **Taxon:** scientificName: Ametadoria leticiamartinezae; phylum: Arthropoda; class: Insecta; order: Diptera; family: Tachinidae; genus: Ametadoria; specificEpithet: leticiamartinezae; scientificNameAuthorship: Fleming & Wood, 2015; **Location:** continent: Central America; country: Costa Rica; countryCode: CR; stateProvince: Guanacaste; county: Area de Conservación Guanacaste; locality: Sector Santa Elena; verbatimLocality: Vado Quebrada Calera; verbatimElevation: 305; verbatimLatitude: 10.867; verbatimLongitude: -85.647; verbatimCoordinateSystem: Decimal; decimalLatitude: 10.867; decimalLongitude: -85.647; **Identification:** identifiedBy: Fleming & Wood; dateIdentified: 2014; **Event:** samplingProtocol: reared from caterpillar of Lactura subfervensDHJ01 (Lacturidae); verbatimEventDate: 12-Aug-2002; **Record Level:** language: en; institutionCode: CNC; collectionCode: Insects; basisOfRecord: Pinned Specimen
- **Type status:** Paratype. **Occurrence:** occurrenceDetails: http://janzen.sas.upenn.edu; catalogNumber: DHJPAR0018983; recordedBy: D.H. Janzen, W. Hallwachs & gusaneros; individualID: DHJPAR0018983; individualCount: 1; sex: F; lifeStage: adult; preparations: pinned; otherCatalogNumbers: 96-SRNP-136; **Taxon:** scientificName: Ametadoria leticiamartinezae; phylum: Arthropoda; class: Insecta; order: Diptera; family: Tachinidae; genus: Ametadoria; specificEpithet: leticiamartinezae; scientificNameAuthorship: Fleming & Wood, 2015; **Location:** continent: Central America; country: Costa Rica; countryCode: CR; stateProvince: Guanacaste; county: Area de Conservación Guanacaste; locality: Sector Murcielago; verbatimLocality: Pozo Del General; verbatimElevation: 40; verbatimLatitude: 10.898; verbatimLongitude: -85.731; verbatimCoordinateSystem: Decimal; decimalLatitude: 10.898; decimalLongitude: -85.731; **Identification:** identifiedBy: Fleming & Wood; dateIdentified: 2014; **Event:** samplingProtocol: reared from caterpillar of Lactura subfervensDHJ01 (Lacturidae); verbatimEventDate: 11-Mar-1996; **Record Level:** language: en; institutionCode: CNC; collectionCode: Insects; basisOfRecord: Pinned Specimen
- **Type status:** Paratype. **Occurrence:** occurrenceDetails: http://janzen.sas.upenn.edu; catalogNumber: DHJPAR0018975; recordedBy: D.H. Janzen, W. Hallwachs & gusaneros; individualID: DHJPAR0018975; individualCount: 1; sex: F; lifeStage: adult; preparations: pinned; otherCatalogNumbers: 02-SRNP-11306; **Taxon:** scientificName: Ametadoria leticiamartinezae; phylum: Arthropoda; class: Insecta; order: Diptera; family: Tachinidae; genus: Ametadoria; specificEpithet: leticiamartinezae; scientificNameAuthorship: Fleming & Wood, 2015; **Location:** continent: Central America; country: Costa Rica; countryCode: CR; stateProvince: Guanacaste; county: Area de Conservación Guanacaste; locality: Sector Santa Elena; verbatimLocality: Vado Quebrada Calera; verbatimElevation: 305; verbatimLatitude: 10.867; verbatimLongitude: -85.647; verbatimCoordinateSystem: Decimal; decimalLatitude: 10.867; decimalLongitude: -85.647; **Identification:** identifiedBy: Fleming & Wood; dateIdentified: 2014; **Event:** samplingProtocol: reared from caterpillar of Lactura subfervensDHJ01 (Lacturidae); verbatimEventDate: 26-Jul-2002; **Record Level:** language: en; institutionCode: CNC; collectionCode: Insects; basisOfRecord: Pinned Specimen
- **Type status:** Paratype. **Occurrence:** occurrenceDetails: http://janzen.sas.upenn.edu; catalogNumber: DHJPAR0018976; recordedBy: D.H. Janzen, W. Hallwachs & gusaneros; individualID: DHJPAR0018976; individualCount: 1; sex: M; lifeStage: adult; preparations: pinned; otherCatalogNumbers: 02-SRNP-11312; **Taxon:** scientificName: Ametadoria leticiamartinezae; phylum: Arthropoda; class: Insecta; order: Diptera; family: Tachinidae; genus: Ametadoria; specificEpithet: leticiamartinezae; scientificNameAuthorship: Fleming & Wood, 2015; **Location:** continent: Central America; country: Costa Rica; countryCode: CR; stateProvince: Guanacaste; county: Area de Conservación Guanacaste; locality: Sector Santa Elena; verbatimLocality: Vado Quebrada Calera; verbatimElevation: 305; verbatimLatitude: 10.867; verbatimLongitude: -85.647; verbatimCoordinateSystem: Decimal; decimalLatitude: 10.867; decimalLongitude: -85.647; **Identification:** identifiedBy: Fleming & Wood; dateIdentified: 2014; **Event:** samplingProtocol: reared from caterpillar of Lactura subfervensDHJ01 (Lacturidae); verbatimEventDate: 04-Aug-2002; **Record Level:** language: en; institutionCode: CNC; collectionCode: Insects; basisOfRecord: Pinned Specimen
- **Type status:** Paratype. **Occurrence:** occurrenceDetails: http://janzen.sas.upenn.edu; catalogNumber: DHJPAR0018985; recordedBy: D.H. Janzen, W. Hallwachs & gusaneros; individualID: DHJPAR0018985; individualCount: 1; sex: M; lifeStage: adult; preparations: pinned; otherCatalogNumbers: 96-SRNP-317; **Taxon:** scientificName: Ametadoria leticiamartinezae; phylum: Arthropoda; class: Insecta; order: Diptera; family: Tachinidae; genus: Ametadoria; specificEpithet: leticiamartinezae; scientificNameAuthorship: Fleming & Wood, 2015; **Location:** continent: Central America; country: Costa Rica; countryCode: CR; stateProvince: Guanacaste; county: Area de Conservación Guanacaste; locality: Sector Murcielago; verbatimLocality: Pozo Del General; verbatimElevation: 40; verbatimLatitude: 10.898; verbatimLongitude: -85.731; verbatimCoordinateSystem: Decimal; decimalLatitude: 10.898; decimalLongitude: -85.731; **Identification:** identifiedBy: Fleming & Wood; dateIdentified: 2014; **Event:** samplingProtocol: reared from caterpillar of Lactura subfervensDHJ01 (Lacturidae); verbatimEventDate: 31-May-1996; **Record Level:** language: en; institutionCode: CNC; collectionCode: Insects; basisOfRecord: Pinned Specimen
- **Type status:** Paratype. **Occurrence:** occurrenceDetails: http://janzen.sas.upenn.edu; catalogNumber: DHJPAR0018984; recordedBy: D.H. Janzen, W. Hallwachs & gusaneros; individualID: DHJPAR0018984; individualCount: 1; sex: F; lifeStage: adult; preparations: pinned; otherCatalogNumbers: 96-SRNP-158; **Taxon:** scientificName: Ametadoria leticiamartinezae; phylum: Arthropoda; class: Insecta; order: Diptera; family: Tachinidae; genus: Ametadoria; specificEpithet: leticiamartinezae; scientificNameAuthorship: Fleming & Wood, 2015; **Location:** continent: Central America; country: Costa Rica; countryCode: CR; stateProvince: Guanacaste; county: Area de Conservación Guanacaste; locality: Sector Murcielago; verbatimLocality: Pozo Del General; verbatimElevation: 40; verbatimLatitude: 10.898; verbatimLongitude: -85.731; verbatimCoordinateSystem: Decimal; decimalLatitude: 10.898; decimalLongitude: -85.731; **Identification:** identifiedBy: Fleming & Wood; dateIdentified: 2014; **Event:** samplingProtocol: reared from caterpillar of Lactura subfervensDHJ01 (Lacturidae); verbatimEventDate: 10-Mar-1996; **Record Level:** language: en; institutionCode: CNC; collectionCode: Insects; basisOfRecord: Pinned Specimen
- **Type status:** Paratype. **Occurrence:** occurrenceDetails: http://janzen.sas.upenn.edu; catalogNumber: DHJPAR0018972; recordedBy: D.H. Janzen, W. Hallwachs & gusaneros; individualID: DHJPAR0018972; individualCount: 1; sex: F; lifeStage: adult; preparations: pinned; otherCatalogNumbers: 02-SRNP-11328; **Taxon:** scientificName: Ametadoria leticiamartinezae; phylum: Arthropoda; class: Insecta; order: Diptera; family: Tachinidae; genus: Ametadoria; specificEpithet: leticiamartinezae; scientificNameAuthorship: Fleming & Wood, 2015; **Location:** continent: Central America; country: Costa Rica; countryCode: CR; stateProvince: Guanacaste; county: Area de Conservación Guanacaste; locality: Sector Santa Elena; verbatimLocality: Vado Quebrada Calera; verbatimElevation: 305; verbatimLatitude: 10.867; verbatimLongitude: -85.647; verbatimCoordinateSystem: Decimal; decimalLatitude: 10.867; decimalLongitude: -85.647; **Identification:** identifiedBy: Fleming & Wood; dateIdentified: 2014; **Event:** samplingProtocol: reared from caterpillar of Lactura subfervensDHJ01 (Lacturidae); verbatimEventDate: 13-Aug-2002; **Record Level:** language: en; institutionCode: CNC; collectionCode: Insects; basisOfRecord: Pinned Specimen

#### Description

**Male** (Fig. 3a, b, c). **Head** (Fig. 3c): frontal vitta dark black, narrowed apically to less than the width of the ocellar triangle, fronto-orbital plate as wide as frontal vitta; ocellar bristles well developed, proclinate, half as long as arista; frontal bristles extending below level of arista by width of one bristle; small black setulae intermingled with frontal bristles all along the parafrontal, not reaching below upper margin of pedicel; antenna black; arista black, minutely pubescent at its base, and bare at its tip; proclinate orbital bristles absent; parafrontal entirely silver; parafacial silver; palpus yellow and haired at its tip; gena 1/7 height of head. **Thorax** (Fig. 3a, b): gray when viewed dorsally with four longitudinal gray vittae, these only slightly visible post-suturally, appearing broken at thoracic suture; four post-sutural dorsocentral bristles; scutellum bearing silver tomentosity over its entirety (this tomentosity extending to underside of scutellum). Legs black; anteroventral surface of hind femur bearing 5–6 long erect bristles of irregular length and irregularly spaced; all surfaces of hind tibia with tightly adpressed, short, regularly spaced hairs, 1 bristle on each of the anterodorsal, anteroventral, posterodorsal and posteroventral surfaces of the tibia, 4X as long as the tibia is wide. Wing (Fig. 3a): pale smoky yellowish in color, calypters pale white translucent, slightly yellow and haired along their margins. **Abdomen** (Fig. 3a): ground color of abdominal tergites brown dorsally overall, dark velvety black medially with a bright gray band covering 3/4 or more of tergal surface. Tergites 3, 4 and 5 possessing median marginal bristles, but syntergite 1+2 without such bristles. Ventrolateral surface of syntergite 1+2, tg 3, and tg 4 orange. Sex patches present on ventral surface of tg 4, and tg 5. **Terminalia** (Figs 5b, 6b, 7b): sternite 5 with a deep and wide V-shaped median cleft, forming two rounded outer lobes, these covered in dense tomentum; lobes of sternite 5 bearing 5 long bristles marginally, the longest approximately 2X as long as other bristles present on sternite 5. Cerci almost flat when viewed laterally, narrow and finger-like in posterior view, not fused medially, maintaining an almost equidistant separation along their entire length; cerci covered in long hair dorsally, tapering at around 4/5 the length of the appendage, leaving only the apex bare. Surstylus short and truncate, approximately 2/3 as long as cerci, rounded and sub-triangular with a slight hook to the tip when viewed laterally; lateral surface of surstylus with several stout bristles confined to anterior apex, these being much stouter and thicker than any other bristles present on the genitalia. Epandrium, when viewed laterally, not extending backwards, 2/3 the length of cerci. Phallus short and stout, approximately same length as cerci. Postgonite 1/3 as long as phalloapodeme with no forward curve, and with a slightly lobed tip.

**Female** (Fig. 3d, e, f). **Head** (Fig. 3f): frontal vitta dark black, narrowed apically to less than the width of the ocellar triangle, fronto-orbital plate as wide as frontal vitta; frontal bristles not reaching below level of pedicel; 2 proclinate orbital bristles present; parafrontal entirely silver with minute setulae over its entire surface; parafacial silver; palpi orange and haired. **Thorax** (Fig. 3d, e): gray when viewed dorsally with four longitudinal gray vittae, these only slightly visible post-suturally, appearing broken at thoracic suture; scutellum bearing silver tomentosity over its entirety; legs and wings as in males. **Abdomen** (Fig. 3d​): abdominal tergites dark velvety black medially, with a bright gray band covering 3/4 or more of tergal surface. Tergites 3, 4 and 5 possessing median marginal bristles, but syntergite 1+2 lacking such bristles.

#### Diagnosis

Fronto-orbital plate without golden tomentum, or golden tomentum present only in trace amounts and directly adjacent to the ocellar triangle; parafacial silver; calypters pale white translucent with yellow margins; hind tibia lacking a regularly spaced comb of hairs; instead, hairs on tibia are tightly adpressed with a few sparse long spines; abdominal tergites with black ground color bearing bright gray tomentose bands covering 3/4 or more of tergal surface; syntergite 1+2 lacking median marginal bristles. Epandrium when viewed laterally not extending backwards, 2/3 length of cerci. Phallus short and stout, approximately same length as cerci. Postgonite 1/3 as long as phalloapodeme with no forward curve, resembling a slightly lobed tip. *Ametadoria leticiamartinezae* can be differentiated from all other species of *Ametadoria* by the following ​combination of traits: the lack of golden tomentum on the fronto-orbital plate, the presence of orange ground color on the ventrolateral surface of the abdomen, and the gray tomentosity of the abdomen.

#### Etymology

*Ametadoria leticiamartinezae* is named in recognition of Ana Leticia Martínez Eras for her contributions to the accounting team for Area de Conservación Guanacaste, the forest this fly lives in.

#### Distribution

Costa Rica, ACG, Prov. Guanacaste, dry forest, 40–305 m elevation.

#### Ecology

Host: *Ametadoria leticiamartinezae* was reared from caterpillars of *Lactura subfervens* (Lacturidae) feeding on *Sideroxylon*​ spp. (Sapotaceae).

### Ametadoria mauriciogurdiani

Fleming & Wood sp. n.

urn:lsid:zoobank.org:act:EBBBDDA0-799B-4C21-94E1-4D9CE5355AD3

#### Materials

- **Type status:** Holotype. **Occurrence:** occurrenceDetails: http://janzen.sas.upenn.edu; catalogNumber: DHJPAR0045547; recordedBy: D.H. Janzen, W. Hallwachs & Roster Moraga; individualID: DHJPAR0045547; individualCount: 1; sex: M; lifeStage: adult; preparations: pinned; otherCatalogNumbers: 11-SRNP-22573; **Taxon:** scientificName: Ametadoria mauriciogurdiani; phylum: Arthropoda; class: Insecta; order: Diptera; family: Tachinidae; genus: Ametadoria; specificEpithet: mauriciogurdiani; scientificNameAuthorship: Fleming & Wood, 2015; **Location:** continent: Central America; country: Costa Rica; countryCode: CR; stateProvince: Guanacaste; county: Area de Conservación Guanacaste; locality: Sector El Hacha; verbatimLocality: Sendero Congos; verbatimElevation: 290; verbatimLatitude: 11.021; verbatimLongitude: -85.525; verbatimCoordinateSystem: Decimal; decimalLatitude: 11.021; decimalLongitude: -85.525; **Identification:** identifiedBy: Fleming & Wood; dateIdentified: 2014; **Event:** samplingProtocol: reared from caterpillar of unidentified Zygaenidae; verbatimEventDate: 22-Oct-2011; **Record Level:** language: en; institutionCode: CNC; collectionCode: Insects; basisOfRecord: Pinned Specimen
- **Type status:** Paratype. **Occurrence:** occurrenceDetails: http://janzen.sas.upenn.edu; catalogNumber: DHJPAR0030182; recordedBy: D.H. Janzen, W. Hallwachs & Manuel Rios; individualID: DHJPAR0030182; individualCount: 1; sex: M; lifeStage: adult; preparations: pinned; otherCatalogNumbers: 08-SRNP-32398; **Taxon:** scientificName: Ametadoria mauriciogurdiani; phylum: Arthropoda; class: Insecta; order: Diptera; family: Tachinidae; genus: Ametadoria; specificEpithet: mauriciogurdiani; scientificNameAuthorship: Fleming & Wood, 2015; **Location:** continent: Central America; country: Costa Rica; countryCode: CR; stateProvince: Guanacaste; county: Area de Conservación Guanacaste; locality: Sector Pitilla; verbatimLocality: Pasmompa; verbatimElevation: 440; verbatimLatitude: 11.019; verbatimLongitude: -85.41; verbatimCoordinateSystem: Decimal; decimalLatitude: 11.019; decimalLongitude: -85.41; **Identification:** identifiedBy: Fleming & Wood; dateIdentified: 2014; **Event:** samplingProtocol: reared from caterpillar of unidentified Zygaenidae; verbatimEventDate: 03-Nov-2008; **Record Level:** language: en; institutionCode: CNC; collectionCode: Insects; basisOfRecord: Pinned Specimen
- **Type status:** Paratype. **Occurrence:** occurrenceDetails: http://janzen.sas.upenn.edu; catalogNumber: DHJPAR0045550; recordedBy: D.H. Janzen, W. Hallwachs & Roster Moraga; individualID: DHJPAR0045550; individualCount: 1; sex: M; lifeStage: adult; preparations: pinned; otherCatalogNumbers: 11-SRNP-22547; **Taxon:** scientificName: Ametadoria mauriciogurdiani; phylum: Arthropoda; class: Insecta; order: Diptera; family: Tachinidae; genus: Ametadoria; specificEpithet: mauriciogurdiani; scientificNameAuthorship: Fleming & Wood, 2015; **Location:** continent: Central America; country: Costa Rica; countryCode: CR; stateProvince: Guanacaste; county: Area de Conservación Guanacaste; locality: Sector El Hacha; verbatimLocality: Sendero Congos; verbatimElevation: 290; verbatimLatitude: 11.021; verbatimLongitude: -85.525; verbatimCoordinateSystem: Decimal; decimalLatitude: 11.021; decimalLongitude: -85.525; **Identification:** identifiedBy: Fleming & Wood; dateIdentified: 2014; **Event:** samplingProtocol: reared from caterpillar of unidentified Zygaenidae; verbatimEventDate: 25-Oct-2011; **Record Level:** language: en; institutionCode: CNC; collectionCode: Insects; basisOfRecord: Pinned Specimen
- **Type status:** Paratype. **Occurrence:** occurrenceDetails: http://janzen.sas.upenn.edu; catalogNumber: DHJPAR0049512; recordedBy: D.H. Janzen, W. Hallwachs & Cirilo Umana; individualID: DHJPAR0049512; individualCount: 1; sex: M; lifeStage: adult; preparations: pinned; otherCatalogNumbers: 12-SRNP-76104; **Taxon:** scientificName: Ametadoria mauriciogurdiani; phylum: Arthropoda; class: Insecta; order: Diptera; family: Tachinidae; genus: Ametadoria; specificEpithet: mauriciogurdiani; scientificNameAuthorship: Fleming & Wood, 2015; **Location:** continent: Central America; country: Costa Rica; countryCode: CR; stateProvince: Guanacaste; county: Area de Conservación Guanacaste; locality: Sector Rincon Rain Forest; verbatimLocality: Finca Esmeralda; verbatimCoordinateSystem: Decimal; **Identification:** identifiedBy: Fleming & Wood; dateIdentified: 2014; **Event:** samplingProtocol: reared from caterpillar of unidentified Zygaenidae; **Record Level:** language: en; institutionCode: CNC; collectionCode: Insects; basisOfRecord: Pinned Specimen
- **Type status:** Paratype. **Occurrence:** occurrenceDetails: http://janzen.sas.upenn.edu; catalogNumber: DHJPAR0030140; recordedBy: D.H. Janzen, W. Hallwachs & Calixto Moraga; individualID: DHJPAR0030140; individualCount: 1; sex: F; lifeStage: adult; preparations: pinned; otherCatalogNumbers: 08-SRNP-32932; **Taxon:** scientificName: Ametadoria mauriciogurdiani; phylum: Arthropoda; class: Insecta; order: Diptera; family: Tachinidae; genus: Ametadoria; specificEpithet: mauriciogurdiani; scientificNameAuthorship: Fleming & Wood, 2015; **Location:** continent: Central America; country: Costa Rica; countryCode: CR; stateProvince: Guanacaste; county: Area de Conservación Guanacaste; locality: Sector Pitilla; verbatimLocality: Estacion Pitilla; verbatimElevation: 675; verbatimLatitude: 10.989; verbatimLongitude: -85.426; verbatimCoordinateSystem: Decimal; decimalLatitude: 10.989; decimalLongitude: -85.426; **Identification:** identifiedBy: Fleming & Wood; dateIdentified: 2014; **Event:** samplingProtocol: reared from caterpillar of unidentified Zygaenidae; verbatimEventDate: 09-Jan-2009; **Record Level:** language: en; institutionCode: CNC; collectionCode: Insects; basisOfRecord: Pinned Specimen
- **Type status:** Paratype. **Occurrence:** occurrenceDetails: http://janzen.sas.upenn.edu; catalogNumber: DHJPAR0045543; recordedBy: D.H. Janzen, W. Hallwachs & Roster Moraga; individualID: DHJPAR0045543; individualCount: 1; sex: F; lifeStage: adult; preparations: pinned; otherCatalogNumbers: 11-SRNP-22568; **Taxon:** scientificName: Ametadoria mauriciogurdiani; phylum: Arthropoda; class: Insecta; order: Diptera; family: Tachinidae; genus: Ametadoria; specificEpithet: mauriciogurdiani; scientificNameAuthorship: Fleming & Wood, 2015; **Location:** continent: Central America; country: Costa Rica; countryCode: CR; stateProvince: Guanacaste; county: Area de Conservación Guanacaste; locality: Sector El Hacha; verbatimLocality: Sendero Congos; verbatimElevation: 290; verbatimLatitude: 11.021; verbatimLongitude: -85.525; verbatimCoordinateSystem: Decimal; decimalLatitude: 11.021; decimalLongitude: -85.525; **Identification:** identifiedBy: Fleming & Wood; dateIdentified: 2014; **Event:** samplingProtocol: reared from caterpillar of unidentified Zygaenidae; verbatimEventDate: 25-Oct-2011; **Record Level:** language: en; institutionCode: CNC; collectionCode: Insects; basisOfRecord: Pinned Specimen
- **Type status:** Paratype. **Occurrence:** occurrenceDetails: http://janzen.sas.upenn.edu; catalogNumber: DHJPAR0018986; recordedBy: D.H. Janzen, W. Hallwachs & gusaneros; individualID: DHJPAR0018986; individualCount: 1; sex: F; lifeStage: adult; preparations: pinned; otherCatalogNumbers: 97-SRNP-5443; **Taxon:** scientificName: Ametadoria mauriciogurdiani; phylum: Arthropoda; class: Insecta; order: Diptera; family: Tachinidae; genus: Ametadoria; specificEpithet: mauriciogurdiani; scientificNameAuthorship: Fleming & Wood, 2015; **Location:** continent: Central America; country: Costa Rica; countryCode: CR; stateProvince: Guanacaste; county: Area de Conservación Guanacaste; locality: Sector El Hacha; verbatimLocality: Sendero Tigre; verbatimElevation: 280; verbatimLatitude: 11.032; verbatimLongitude: -85.526; verbatimCoordinateSystem: Decimal; decimalLatitude: 11.032; decimalLongitude: -85.526; **Identification:** identifiedBy: Fleming & Wood; dateIdentified: 2014; **Event:** samplingProtocol: reared from caterpillar of unidentified Zygaenidae; verbatimEventDate: 31-Oct-1997; **Record Level:** language: en; institutionCode: CNC; collectionCode: Insects; basisOfRecord: Pinned Specimen
- **Type status:** Paratype. **Occurrence:** occurrenceDetails: http://janzen.sas.upenn.edu; catalogNumber: DHJPAR0035727; recordedBy: D.H. Janzen, W. Hallwachs & Cirilo Umana; individualID: DHJPAR0035727; individualCount: 1; sex: F; lifeStage: adult; preparations: pinned; otherCatalogNumbers: 09-SRNP-44881; **Taxon:** scientificName: Ametadoria mauriciogurdiani; phylum: Arthropoda; class: Insecta; order: Diptera; family: Tachinidae; genus: Ametadoria; specificEpithet: mauriciogurdiani; scientificNameAuthorship: Fleming & Wood, 2015; **Location:** continent: Central America; country: Costa Rica; countryCode: CR; stateProvince: Alajuela; county: Area de Conservación Guanacaste; locality: Sector Rincon Rain Forest; verbatimLocality: Estacion Llanura; verbatimElevation: 135; verbatimLatitude: 10.933; verbatimLongitude: -85.253; verbatimCoordinateSystem: Decimal; decimalLatitude: 10.933; decimalLongitude: -85.253; **Identification:** identifiedBy: Fleming & Wood; dateIdentified: 2014; **Event:** samplingProtocol: reared from caterpillar of unidentified Zygaenidae; verbatimEventDate: 08-Mar-2009; **Record Level:** language: en; institutionCode: CNC; collectionCode: Insects; basisOfRecord: Pinned Specimen
- **Type status:** Paratype. **Occurrence:** occurrenceDetails: http://janzen.sas.upenn.edu; catalogNumber: DHJPAR0049504; recordedBy: D.H. Janzen, W. Hallwachs & Cirilo Umana; individualID: DHJPAR0049504; individualCount: 1; sex: F; lifeStage: adult; preparations: pinned; otherCatalogNumbers: 12-SRNP-76113; **Taxon:** scientificName: Ametadoria mauriciogurdiani; phylum: Arthropoda; class: Insecta; order: Diptera; family: Tachinidae; genus: Ametadoria; specificEpithet: mauriciogurdiani; scientificNameAuthorship: Fleming & Wood, 2015; **Location:** continent: Central America; country: Costa Rica; countryCode: CR; stateProvince: Guanacaste; county: Area de Conservación Guanacaste; locality: Sector Rincon Rain Forest; verbatimLocality: Finca Esmeralda; verbatimCoordinateSystem: Decimal; **Identification:** identifiedBy: Fleming & Wood; dateIdentified: 2014; **Event:** samplingProtocol: reared from caterpillar of unidentified Zygaenidae; **Record Level:** language: en; institutionCode: CNC; collectionCode: Insects; basisOfRecord: Pinned Specimen
- **Type status:** Paratype. **Occurrence:** occurrenceDetails: http://janzen.sas.upenn.edu; catalogNumber: DHJPAR0045549; recordedBy: D.H. Janzen, W. Hallwachs & Roster Moraga; individualID: DHJPAR0045549; individualCount: 1; sex: M; lifeStage: adult; preparations: pinned; otherCatalogNumbers: 11-SRNP-22556; **Taxon:** scientificName: Ametadoria mauriciogurdiani; phylum: Arthropoda; class: Insecta; order: Diptera; family: Tachinidae; genus: Ametadoria; specificEpithet: mauriciogurdiani; scientificNameAuthorship: Fleming & Wood, 2015; **Location:** continent: Central America; country: Costa Rica; countryCode: CR; stateProvince: Guanacaste; county: Area de Conservación Guanacaste; locality: Sector El Hacha; verbatimLocality: Sendero Congos; verbatimElevation: 290; verbatimLatitude: 11.021; verbatimLongitude: -85.525; verbatimCoordinateSystem: Decimal; decimalLatitude: 11.021; decimalLongitude: -85.525; **Identification:** identifiedBy: Fleming & Wood; dateIdentified: 2014; **Event:** samplingProtocol: reared from caterpillar of unidentified Zygaenidae; verbatimEventDate: 22-Oct-2011; **Record Level:** language: en; institutionCode: CNC; collectionCode: Insects; basisOfRecord: Pinned Specimen
- **Type status:** Paratype. **Occurrence:** occurrenceDetails: http://janzen.sas.upenn.edu; catalogNumber: DHJPAR0007041; recordedBy: D.H. Janzen, W. Hallwachs & Petrona Rios; individualID: DHJPAR0007041; individualCount: 1; sex: M; lifeStage: adult; preparations: pinned; otherCatalogNumbers: 05-SRNP-34130; **Taxon:** scientificName: Ametadoria mauriciogurdiani; phylum: Arthropoda; class: Insecta; order: Diptera; family: Tachinidae; genus: Ametadoria; specificEpithet: mauriciogurdiani; scientificNameAuthorship: Fleming & Wood, 2015; **Location:** continent: Central America; country: Costa Rica; countryCode: CR; stateProvince: Guanacaste; county: Area de Conservación Guanacaste; locality: Sector Pitilla; verbatimLocality: Pasmompa; verbatimElevation: 440; verbatimLatitude: 11.019; verbatimLongitude: -85.41; verbatimCoordinateSystem: Decimal; decimalLatitude: 11.019; decimalLongitude: -85.41; **Identification:** identifiedBy: Fleming & Wood; dateIdentified: 2014; **Event:** samplingProtocol: reared from caterpillar of unidentified Zygaenidae; verbatimEventDate: 12-Nov-2005; **Record Level:** language: en; institutionCode: CNC; collectionCode: Insects; basisOfRecord: Pinned Specimen
- **Type status:** Paratype. **Occurrence:** occurrenceDetails: http://janzen.sas.upenn.edu; catalogNumber: DHJPAR0030143; recordedBy: D.H. Janzen, W. Hallwachs & Calixto Moraga; individualID: DHJPAR0030143; individualCount: 1; sex: M; lifeStage: adult; preparations: pinned; otherCatalogNumbers: 08-SRNP-32881; **Taxon:** scientificName: Ametadoria mauriciogurdiani; phylum: Arthropoda; class: Insecta; order: Diptera; family: Tachinidae; genus: Ametadoria; specificEpithet: mauriciogurdiani; scientificNameAuthorship: Fleming & Wood, 2015; **Location:** continent: Central America; country: Costa Rica; countryCode: CR; stateProvince: Guanacaste; county: Area de Conservación Guanacaste; locality: Sector Pitilla; verbatimLocality: Estacion Pitilla; verbatimElevation: 675; verbatimLatitude: 10.989; verbatimLongitude: -85.426; verbatimCoordinateSystem: Decimal; decimalLatitude: 10.989; decimalLongitude: -85.426; **Identification:** identifiedBy: Fleming & Wood; dateIdentified: 2014; **Event:** samplingProtocol: reared from caterpillar of unidentified Zygaenidae; verbatimEventDate: 13-Jan-2009; **Record Level:** language: en; institutionCode: CNC; collectionCode: Insects; basisOfRecord: Pinned Specimen
- **Type status:** Paratype. **Occurrence:** occurrenceDetails: http://janzen.sas.upenn.edu; catalogNumber: DHJPAR0034407; recordedBy: D.H. Janzen, W. Hallwachs & Cirilo Umana; individualID: DHJPAR0034407; individualCount: 1; sex: M; lifeStage: adult; preparations: pinned; otherCatalogNumbers: 09-SRNP-44358; **Taxon:** scientificName: Ametadoria mauriciogurdiani; phylum: Arthropoda; class: Insecta; order: Diptera; family: Tachinidae; genus: Ametadoria; specificEpithet: mauriciogurdiani; scientificNameAuthorship: Fleming & Wood, 2015; **Location:** continent: Central America; country: Costa Rica; countryCode: CR; stateProvince: Alajuela; county: Area de Conservación Guanacaste; locality: Sector Rincon Rain Forest; verbatimLocality: Estacion Llanura; verbatimElevation: 135; verbatimLatitude: 10.933; verbatimLongitude: -85.253; verbatimCoordinateSystem: Decimal; decimalLatitude: 10.933; decimalLongitude: -85.253; **Identification:** identifiedBy: Fleming & Wood; dateIdentified: 2014; **Event:** samplingProtocol: reared from caterpillar of unidentified Zygaenidae; verbatimEventDate: 15-Jun-2009; **Record Level:** language: en; institutionCode: CNC; collectionCode: Insects; basisOfRecord: Pinned Specimen
- **Type status:** Paratype. **Occurrence:** occurrenceDetails: http://janzen.sas.upenn.edu; catalogNumber: DHJPAR0045545; recordedBy: D.H. Janzen, W. Hallwachs & Roster Moraga; individualID: DHJPAR0045545; individualCount: 1; sex: M; lifeStage: adult; preparations: pinned; otherCatalogNumbers: 11-SRNP-22553; **Taxon:** scientificName: Ametadoria mauriciogurdiani; phylum: Arthropoda; class: Insecta; order: Diptera; family: Tachinidae; genus: Ametadoria; specificEpithet: mauriciogurdiani; scientificNameAuthorship: Fleming & Wood, 2015; **Location:** continent: Central America; country: Costa Rica; countryCode: CR; stateProvince: Guanacaste; county: Area de Conservación Guanacaste; locality: Sector El Hacha; verbatimLocality: Sendero Congos; verbatimElevation: 290; verbatimLatitude: 11.021; verbatimLongitude: -85.525; verbatimCoordinateSystem: Decimal; decimalLatitude: 11.021; decimalLongitude: -85.525; **Identification:** identifiedBy: Fleming & Wood; dateIdentified: 2014; **Event:** samplingProtocol: reared from caterpillar of unidentified Zygaenidae; verbatimEventDate: 25-Oct-2011; **Record Level:** language: en; institutionCode: CNC; collectionCode: Insects; basisOfRecord: Pinned Specimen
- **Type status:** Paratype. **Occurrence:** occurrenceDetails: http://janzen.sas.upenn.edu; catalogNumber: DHJPAR0035725; recordedBy: D.H. Janzen, W. Hallwachs & Cirilo Umana; individualID: DHJPAR0035725; individualCount: 1; sex: M; lifeStage: adult; preparations: pinned; otherCatalogNumbers: 09-SRNP-44882; **Taxon:** scientificName: Ametadoria mauriciogurdiani; phylum: Arthropoda; class: Insecta; order: Diptera; family: Tachinidae; genus: Ametadoria; specificEpithet: mauriciogurdiani; scientificNameAuthorship: Fleming & Wood, 2015; **Location:** continent: Central America; country: Costa Rica; countryCode: CR; stateProvince: Alajuela; county: Area de Conservación Guanacaste; locality: Sector Rincon Rain Forest; verbatimLocality: Estacion Llanura; verbatimElevation: 135; verbatimLatitude: 10.933; verbatimLongitude: -85.253; verbatimCoordinateSystem: Decimal; decimalLatitude: 10.933; decimalLongitude: -85.253; **Identification:** identifiedBy: Fleming & Wood; dateIdentified: 2014; **Event:** samplingProtocol: reared from caterpillar of unidentified Zygaenidae; verbatimEventDate: 31-Jul-2009; **Record Level:** language: en; institutionCode: CNC; collectionCode: Insects; basisOfRecord: Pinned Specimen
- **Type status:** Paratype. **Occurrence:** occurrenceDetails: http://janzen.sas.upenn.edu; catalogNumber: DHJPAR0030141; recordedBy: D.H. Janzen, W. Hallwachs & Calixto Moraga; individualID: DHJPAR0030141; individualCount: 1; sex: F; lifeStage: adult; preparations: pinned; otherCatalogNumbers: 08-SRNP-32878; **Taxon:** scientificName: Ametadoria mauriciogurdiani; phylum: Arthropoda; class: Insecta; order: Diptera; family: Tachinidae; genus: Ametadoria; specificEpithet: mauriciogurdiani; scientificNameAuthorship: Fleming & Wood, 2015; **Location:** continent: Central America; country: Costa Rica; countryCode: CR; stateProvince: Guanacaste; county: Area de Conservación Guanacaste; locality: Sector Pitilla; verbatimLocality: Estacion Pitilla; verbatimElevation: 675; verbatimLatitude: 10.989; verbatimLongitude: -85.426; verbatimCoordinateSystem: Decimal; decimalLatitude: 10.989; decimalLongitude: -85.426; **Identification:** identifiedBy: Fleming & Wood; dateIdentified: 2014; **Event:** samplingProtocol: reared from caterpillar of unidentified Zygaenidae; verbatimEventDate: 16-Jan-2009; **Record Level:** language: en; institutionCode: CNC; collectionCode: Insects; basisOfRecord: Pinned Specimen
- **Type status:** Paratype. **Occurrence:** occurrenceDetails: http://janzen.sas.upenn.edu; catalogNumber: DHJPAR0045552; recordedBy: D.H. Janzen, W. Hallwachs & Elieth Cantillano; individualID: DHJPAR0045552; individualCount: 1; sex: F; lifeStage: adult; preparations: pinned; otherCatalogNumbers: 11-SRNP-22736; **Taxon:** scientificName: Ametadoria mauriciogurdiani; phylum: Arthropoda; class: Insecta; order: Diptera; family: Tachinidae; genus: Ametadoria; specificEpithet: mauriciogurdiani; scientificNameAuthorship: Fleming & Wood, 2015; **Location:** continent: Central America; country: Costa Rica; countryCode: CR; stateProvince: Guanacaste; county: Area de Conservación Guanacaste; locality: Sector El Hacha; verbatimLocality: Estacion Los Almendros; verbatimElevation: 290; verbatimLatitude: 11.032; verbatimLongitude: -85.528; verbatimCoordinateSystem: Decimal; decimalLatitude: 11.032; decimalLongitude: -85.528; **Identification:** identifiedBy: Fleming & Wood; dateIdentified: 2014; **Event:** samplingProtocol: reared from caterpillar of unidentified Zygaenidae; verbatimEventDate: 25-Oct-2011; **Record Level:** language: en; institutionCode: CNC; collectionCode: Insects; basisOfRecord: Pinned Specimen
- **Type status:** Paratype. **Occurrence:** occurrenceDetails: http://janzen.sas.upenn.edu; catalogNumber: DHJPAR0045548; recordedBy: D.H. Janzen, W. Hallwachs & Roster Moraga; individualID: DHJPAR0045548; individualCount: 1; sex: M; lifeStage: adult; preparations: pinned; otherCatalogNumbers: 11-SRNP-22708; **Taxon:** scientificName: Ametadoria mauriciogurdiani; phylum: Arthropoda; class: Insecta; order: Diptera; family: Tachinidae; genus: Ametadoria; specificEpithet: mauriciogurdiani; scientificNameAuthorship: Fleming & Wood, 2015; **Location:** continent: Central America; country: Costa Rica; countryCode: CR; stateProvince: Guanacaste; county: Area de Conservación Guanacaste; locality: Sector El Hacha; verbatimLocality: Sendero Congos; verbatimElevation: 290; verbatimLatitude: 11.021; verbatimLongitude: -85.525; verbatimCoordinateSystem: Decimal; decimalLatitude: 11.021; decimalLongitude: -85.525; **Identification:** identifiedBy: Fleming & Wood; dateIdentified: 2014; **Event:** samplingProtocol: reared from caterpillar of unidentified Zygaenidae; verbatimEventDate: 22-Oct-2011; **Record Level:** language: en; institutionCode: CNC; collectionCode: Insects; basisOfRecord: Pinned Specimen
- **Type status:** Paratype. **Occurrence:** occurrenceDetails: http://janzen.sas.upenn.edu; catalogNumber: DHJPAR0045632; recordedBy: D.H. Janzen, W. Hallwachs & Freddy Quesada; individualID: DHJPAR0045632; individualCount: 1; sex: F; lifeStage: adult; preparations: pinned; otherCatalogNumbers: 11-SRNP-32601; **Taxon:** scientificName: Ametadoria mauriciogurdiani; phylum: Arthropoda; class: Insecta; order: Diptera; family: Tachinidae; genus: Ametadoria; specificEpithet: mauriciogurdiani; scientificNameAuthorship: Fleming & Wood, 2015; **Location:** continent: Central America; country: Costa Rica; countryCode: CR; stateProvince: Guanacaste; county: Area de Conservación Guanacaste; locality: Sector Pitilla; verbatimLocality: Casa Roberto; verbatimElevation: 520; verbatimLatitude: 11.011; verbatimLongitude: -85.421; verbatimCoordinateSystem: Decimal; decimalLatitude: 11.011; decimalLongitude: -85.421; **Identification:** identifiedBy: Fleming & Wood; dateIdentified: 2014; **Event:** samplingProtocol: reared from caterpillar of unidentified Zygaenidae; verbatimEventDate: 19-Oct-2011; **Record Level:** language: en; institutionCode: CNC; collectionCode: Insects; basisOfRecord: Pinned Specimen
- **Type status:** Paratype. **Occurrence:** occurrenceDetails: http://janzen.sas.upenn.edu; catalogNumber: DHJPAR0035731; recordedBy: D.H. Janzen, W. Hallwachs & Cirilo Umana; individualID: DHJPAR0035731; individualCount: 1; sex: M; lifeStage: adult; preparations: pinned; otherCatalogNumbers: 09-SRNP-44871; **Taxon:** scientificName: Ametadoria mauriciogurdiani; phylum: Arthropoda; class: Insecta; order: Diptera; family: Tachinidae; genus: Ametadoria; specificEpithet: mauriciogurdiani; scientificNameAuthorship: Fleming & Wood, 2015; **Location:** continent: Central America; country: Costa Rica; countryCode: CR; stateProvince: Alajuela; county: Area de Conservación Guanacaste; locality: Sector Rincon Rain Forest; verbatimLocality: Estacion Llanura; verbatimElevation: 135; verbatimLatitude: 10.933; verbatimLongitude: -85.253; verbatimCoordinateSystem: Decimal; decimalLatitude: 10.933; decimalLongitude: -85.253; **Identification:** identifiedBy: Fleming & Wood; dateIdentified: 2014; **Event:** samplingProtocol: reared from caterpillar of unidentified Zygaenidae; verbatimEventDate: 27-Jul-2009; **Record Level:** language: en; institutionCode: CNC; collectionCode: Insects; basisOfRecord: Pinned Specimen
- **Type status:** Paratype. **Occurrence:** occurrenceDetails: http://janzen.sas.upenn.edu; catalogNumber: DHJPAR0030149; recordedBy: D.H. Janzen, W. Hallwachs & Calixto Moraga; individualID: DHJPAR0030149; individualCount: 1; sex: F; lifeStage: adult; preparations: pinned; otherCatalogNumbers: 08-SRNP-32906; **Taxon:** scientificName: Ametadoria mauriciogurdiani; phylum: Arthropoda; class: Insecta; order: Diptera; family: Tachinidae; genus: Ametadoria; specificEpithet: mauriciogurdiani; scientificNameAuthorship: Fleming & Wood, 2015; **Location:** continent: Central America; country: Costa Rica; countryCode: CR; stateProvince: Guanacaste; county: Area de Conservación Guanacaste; locality: Sector Pitilla; verbatimLocality: Estacion Pitilla; verbatimElevation: 675; verbatimLatitude: 10.989; verbatimLongitude: -85.426; verbatimCoordinateSystem: Decimal; decimalLatitude: 10.989; decimalLongitude: -85.426; **Identification:** identifiedBy: Fleming & Wood; dateIdentified: 2014; **Event:** samplingProtocol: reared from caterpillar of unidentified Zygaenidae; verbatimEventDate: 09-Jan-2009; **Record Level:** language: en; institutionCode: CNC; collectionCode: Insects; basisOfRecord: Pinned Specimen
- **Type status:** Paratype. **Occurrence:** occurrenceDetails: http://janzen.sas.upenn.edu; catalogNumber: DHJPAR0045554; recordedBy: D.H. Janzen, W. Hallwachs & Roster Moraga; individualID: DHJPAR0045554; individualCount: 1; sex: M; lifeStage: adult; preparations: pinned; otherCatalogNumbers: 11-SRNP-22570; **Taxon:** scientificName: Ametadoria mauriciogurdiani; phylum: Arthropoda; class: Insecta; order: Diptera; family: Tachinidae; genus: Ametadoria; specificEpithet: mauriciogurdiani; scientificNameAuthorship: Fleming & Wood, 2015; **Location:** continent: Central America; country: Costa Rica; countryCode: CR; stateProvince: Guanacaste; county: Area de Conservación Guanacaste; locality: Sector El Hacha; verbatimLocality: Sendero Congos; verbatimElevation: 290; verbatimLatitude: 11.021; verbatimLongitude: -85.525; verbatimCoordinateSystem: Decimal; decimalLatitude: 11.021; decimalLongitude: -85.525; **Identification:** identifiedBy: Fleming & Wood; dateIdentified: 2014; **Event:** samplingProtocol: reared from caterpillar of unidentified Zygaenidae; verbatimEventDate: 25-Oct-2011; **Record Level:** language: en; institutionCode: CNC; collectionCode: Insects; basisOfRecord: Pinned Specimen
- **Type status:** Paratype. **Occurrence:** occurrenceDetails: http://janzen.sas.upenn.edu; catalogNumber: DHJPAR0030454; recordedBy: D.H. Janzen, W. Hallwachs & Calixto Moraga; individualID: DHJPAR0030454; individualCount: 1; sex: M; lifeStage: adult; preparations: pinned; otherCatalogNumbers: 08-SRNP-33104; **Taxon:** scientificName: Ametadoria mauriciogurdiani; phylum: Arthropoda; class: Insecta; order: Diptera; family: Tachinidae; genus: Ametadoria; specificEpithet: mauriciogurdiani; scientificNameAuthorship: Fleming & Wood, 2015; **Location:** continent: Central America; country: Costa Rica; countryCode: CR; stateProvince: Guanacaste; county: Area de Conservación Guanacaste; locality: Sector Pitilla; verbatimLocality: Estacion Pitilla; verbatimElevation: 675; verbatimLatitude: 10.989; verbatimLongitude: -85.426; verbatimCoordinateSystem: Decimal; decimalLatitude: 10.989; decimalLongitude: -85.426; **Identification:** identifiedBy: Fleming & Wood; dateIdentified: 2014; **Event:** samplingProtocol: reared from caterpillar of unidentified Zygaenidae; verbatimEventDate: 16-Jan-2009; **Record Level:** language: en; institutionCode: CNC; collectionCode: Insects; basisOfRecord: Pinned Specimen
- **Type status:** Paratype. **Occurrence:** occurrenceDetails: http://janzen.sas.upenn.edu; catalogNumber: DHJPAR0030142; recordedBy: D.H. Janzen, W. Hallwachs & Calixto Moraga; individualID: DHJPAR0030142; individualCount: 1; sex: F; lifeStage: adult; preparations: pinned; otherCatalogNumbers: 08-SRNP-32903; **Taxon:** scientificName: Ametadoria mauriciogurdiani; phylum: Arthropoda; class: Insecta; order: Diptera; family: Tachinidae; genus: Ametadoria; specificEpithet: mauriciogurdiani; scientificNameAuthorship: Fleming & Wood, 2015; **Location:** continent: Central America; country: Costa Rica; countryCode: CR; stateProvince: Guanacaste; county: Area de Conservación Guanacaste; locality: Sector Pitilla; verbatimLocality: Estacion Pitilla; verbatimElevation: 675; verbatimLatitude: 10.989; verbatimLongitude: -85.426; verbatimCoordinateSystem: Decimal; decimalLatitude: 10.989; decimalLongitude: -85.426; **Identification:** identifiedBy: Fleming & Wood; dateIdentified: 2014; **Event:** samplingProtocol: reared from caterpillar of unidentified Zygaenidae; verbatimEventDate: 09-Jan-2009; **Record Level:** language: en; institutionCode: CNC; collectionCode: Insects; basisOfRecord: Pinned Specimen
- **Type status:** Paratype. **Occurrence:** occurrenceDetails: http://janzen.sas.upenn.edu; catalogNumber: DHJPAR0037247; recordedBy: D.H. Janzen, W. Hallwachs & Calixto Moraga; individualID: DHJPAR0037247; individualCount: 1; sex: M; lifeStage: adult; preparations: pinned; otherCatalogNumbers: 09-SRNP-32986; **Taxon:** scientificName: Ametadoria mauriciogurdiani; phylum: Arthropoda; class: Insecta; order: Diptera; family: Tachinidae; genus: Ametadoria; specificEpithet: mauriciogurdiani; scientificNameAuthorship: Fleming & Wood, 2015; **Location:** continent: Central America; country: Costa Rica; countryCode: CR; stateProvince: Guanacaste; county: Area de Conservación Guanacaste; locality: Sector Pitilla; verbatimLocality: Estacion Pitilla; verbatimElevation: 675; verbatimLatitude: 10.989; verbatimLongitude: -85.426; verbatimCoordinateSystem: Decimal; decimalLatitude: 10.989; decimalLongitude: -85.426; **Identification:** identifiedBy: Fleming & Wood; dateIdentified: 2014; **Event:** samplingProtocol: reared from caterpillar of unidentified Zygaenidae; verbatimEventDate: 04-Dec-2009; **Record Level:** language: en; institutionCode: CNC; collectionCode: Insects; basisOfRecord: Pinned Specimen
- **Type status:** Paratype. **Occurrence:** occurrenceDetails: http://janzen.sas.upenn.edu; catalogNumber: DHJPAR0045544; recordedBy: D.H. Janzen, W. Hallwachs & Roster Moraga; individualID: DHJPAR0045544; individualCount: 1; sex: F; lifeStage: adult; preparations: pinned; otherCatalogNumbers: 11-SRNP-22571; **Taxon:** scientificName: Ametadoria mauriciogurdiani; phylum: Arthropoda; class: Insecta; order: Diptera; family: Tachinidae; genus: Ametadoria; specificEpithet: mauriciogurdiani; scientificNameAuthorship: Fleming & Wood, 2015; **Location:** continent: Central America; country: Costa Rica; countryCode: CR; stateProvince: Guanacaste; county: Area de Conservación Guanacaste; locality: Sector El Hacha; verbatimLocality: Sendero Congos; verbatimElevation: 290; verbatimLatitude: 11.021; verbatimLongitude: -85.525; verbatimCoordinateSystem: Decimal; decimalLatitude: 11.021; decimalLongitude: -85.525; **Identification:** identifiedBy: Fleming & Wood; dateIdentified: 2014; **Event:** samplingProtocol: reared from caterpillar of unidentified Zygaenidae; verbatimEventDate: 23-Oct-2011; **Record Level:** language: en; institutionCode: CNC; collectionCode: Insects; basisOfRecord: Pinned Specimen
- **Type status:** Paratype. **Occurrence:** occurrenceDetails: http://janzen.sas.upenn.edu; catalogNumber: DHJPAR0037263; recordedBy: D.H. Janzen, W. Hallwachs & Calixto Moraga; individualID: DHJPAR0037263; individualCount: 1; sex: M; lifeStage: adult; preparations: pinned; otherCatalogNumbers: 09-SRNP-32989; **Taxon:** scientificName: Ametadoria mauriciogurdiani; phylum: Arthropoda; class: Insecta; order: Diptera; family: Tachinidae; genus: Ametadoria; specificEpithet: mauriciogurdiani; scientificNameAuthorship: Fleming & Wood, 2015; **Location:** continent: Central America; country: Costa Rica; countryCode: CR; stateProvince: Guanacaste; county: Area de Conservación Guanacaste; locality: Sector Pitilla; verbatimLocality: Estacion Pitilla; verbatimElevation: 675; verbatimLatitude: 10.989; verbatimLongitude: -85.426; verbatimCoordinateSystem: Decimal; decimalLatitude: 10.989; decimalLongitude: -85.426; **Identification:** identifiedBy: Fleming & Wood; dateIdentified: 2014; **Event:** samplingProtocol: reared from caterpillar of unidentified Zygaenidae; verbatimEventDate: 03-Dec-2009; **Record Level:** language: en; institutionCode: CNC; collectionCode: Insects; basisOfRecord: Pinned Specimen
- **Type status:** Paratype. **Occurrence:** occurrenceDetails: http://janzen.sas.upenn.edu; catalogNumber: DHJPAR0045551; recordedBy: D.H. Janzen, W. Hallwachs & Roster Moraga; individualID: DHJPAR0045551; individualCount: 1; sex: F; lifeStage: adult; preparations: pinned; otherCatalogNumbers: 11-SRNP-22574; **Taxon:** scientificName: Ametadoria mauriciogurdiani; phylum: Arthropoda; class: Insecta; order: Diptera; family: Tachinidae; genus: Ametadoria; specificEpithet: mauriciogurdiani; scientificNameAuthorship: Fleming & Wood, 2015; **Location:** continent: Central America; country: Costa Rica; countryCode: CR; stateProvince: Guanacaste; county: Area de Conservación Guanacaste; locality: Sector El Hacha; verbatimLocality: Sendero Congos; verbatimElevation: 290; verbatimLatitude: 11.021; verbatimLongitude: -85.525; verbatimCoordinateSystem: Decimal; decimalLatitude: 11.021; decimalLongitude: -85.525; **Identification:** identifiedBy: Fleming & Wood; dateIdentified: 2014; **Event:** samplingProtocol: reared from caterpillar of unidentified Zygaenidae; verbatimEventDate: 25-Oct-2011; **Record Level:** language: en; institutionCode: CNC; collectionCode: Insects; basisOfRecord: Pinned Specimen
- **Type status:** Paratype. **Occurrence:** occurrenceDetails: http://janzen.sas.upenn.edu; catalogNumber: DHJPAR0035726; recordedBy: D.H. Janzen, W. Hallwachs & Cirilo Umana; individualID: DHJPAR0035726; individualCount: 1; sex: M; lifeStage: adult; preparations: pinned; otherCatalogNumbers: 09-SRNP-44879; **Taxon:** scientificName: Ametadoria mauriciogurdiani; phylum: Arthropoda; class: Insecta; order: Diptera; family: Tachinidae; genus: Ametadoria; specificEpithet: mauriciogurdiani; scientificNameAuthorship: Fleming & Wood, 2015; **Location:** continent: Central America; country: Costa Rica; countryCode: CR; stateProvince: Alajuela; county: Area de Conservación Guanacaste; locality: Sector Rincon Rain Forest; verbatimLocality: Estacion Llanura; verbatimElevation: 135; verbatimLatitude: 10.933; verbatimLongitude: -85.253; verbatimCoordinateSystem: Decimal; decimalLatitude: 10.933; decimalLongitude: -85.253; **Identification:** identifiedBy: Fleming & Wood; dateIdentified: 2014; **Event:** samplingProtocol: reared from caterpillar of unidentified Zygaenidae; verbatimEventDate: 28-Jul-2009; **Record Level:** language: en; institutionCode: CNC; collectionCode: Insects; basisOfRecord: Pinned Specimen
- **Type status:** Paratype. **Occurrence:** occurrenceDetails: http://janzen.sas.upenn.edu; catalogNumber: DHJPAR0035729; recordedBy: D.H. Janzen, W. Hallwachs & Cirilo Umana; individualID: DHJPAR0035729; individualCount: 1; sex: F; lifeStage: adult; preparations: pinned; otherCatalogNumbers: 09-SRNP-44869; **Taxon:** scientificName: Ametadoria mauriciogurdiani; phylum: Arthropoda; class: Insecta; order: Diptera; family: Tachinidae; genus: Ametadoria; specificEpithet: mauriciogurdiani; scientificNameAuthorship: Fleming & Wood, 2015; **Location:** continent: Central America; country: Costa Rica; countryCode: CR; stateProvince: Alajuela; county: Area de Conservación Guanacaste; locality: Sector Rincon Rain Forest; verbatimLocality: Estacion Llanura; verbatimElevation: 135; verbatimLatitude: 10.933; verbatimLongitude: -85.253; verbatimCoordinateSystem: Decimal; decimalLatitude: 10.933; decimalLongitude: -85.253; **Identification:** identifiedBy: Fleming & Wood; dateIdentified: 2014; **Event:** samplingProtocol: reared from caterpillar of unidentified Zygaenidae; verbatimEventDate: 03-Aug-2009; **Record Level:** language: en; institutionCode: CNC; collectionCode: Insects; basisOfRecord: Pinned Specimen
- **Type status:** Paratype. **Occurrence:** occurrenceDetails: http://janzen.sas.upenn.edu; catalogNumber: DHJPAR0045553; recordedBy: D.H. Janzen, W. Hallwachs & Roster Moraga; individualID: DHJPAR0045553; individualCount: 1; sex: F; lifeStage: adult; preparations: pinned; otherCatalogNumbers: 11-SRNP-22577; **Taxon:** scientificName: Ametadoria mauriciogurdiani; phylum: Arthropoda; class: Insecta; order: Diptera; family: Tachinidae; genus: Ametadoria; specificEpithet: mauriciogurdiani; scientificNameAuthorship: Fleming & Wood, 2015; **Location:** continent: Central America; country: Costa Rica; countryCode: CR; stateProvince: Guanacaste; county: Area de Conservación Guanacaste; locality: Sector El Hacha; verbatimLocality: Sendero Congos; verbatimElevation: 290; verbatimLatitude: 11.021; verbatimLongitude: -85.525; verbatimCoordinateSystem: Decimal; decimalLatitude: 11.021; decimalLongitude: -85.525; **Identification:** identifiedBy: Fleming & Wood; dateIdentified: 2014; **Event:** samplingProtocol: reared from caterpillar of unidentified Zygaenidae; verbatimEventDate: 25-Oct-2011; **Record Level:** language: en; institutionCode: CNC; collectionCode: Insects; basisOfRecord: Pinned Specimen
- **Type status:** Paratype. **Occurrence:** occurrenceDetails: http://janzen.sas.upenn.edu; catalogNumber: DHJPAR0030011; recordedBy: D.H. Janzen, W. Hallwachs & Jose Perez; individualID: DHJPAR0030011; individualCount: 1; sex: F; lifeStage: adult; preparations: pinned; otherCatalogNumbers: 08-SRNP-42254; **Taxon:** scientificName: Ametadoria mauriciogurdiani; phylum: Arthropoda; class: Insecta; order: Diptera; family: Tachinidae; genus: Ametadoria; specificEpithet: mauriciogurdiani; scientificNameAuthorship: Fleming & Wood, 2015; **Location:** continent: Central America; country: Costa Rica; countryCode: CR; stateProvince: Alajuela; county: Area de Conservación Guanacaste; locality: Sector Rincon Rain Forest; verbatimLocality: Sendero Tucan; verbatimElevation: 410; verbatimLatitude: 10.904; verbatimLongitude: -85.271; verbatimCoordinateSystem: Decimal; decimalLatitude: 10.904; decimalLongitude: -85.271; **Identification:** identifiedBy: Fleming & Wood; dateIdentified: 2014; **Event:** samplingProtocol: reared from caterpillar of unidentified Zygaenidae; verbatimEventDate: 28-Dec-2008; **Record Level:** language: en; institutionCode: CNC; collectionCode: Insects; basisOfRecord: Pinned Specimen
- **Type status:** Paratype. **Occurrence:** occurrenceDetails: http://janzen.sas.upenn.edu; catalogNumber: DHJPAR0045639; recordedBy: D.H. Janzen, W. Hallwachs & Freddy Quesada; individualID: DHJPAR0045639; individualCount: 1; sex: F; lifeStage: adult; preparations: pinned; otherCatalogNumbers: 11-SRNP-32602; **Taxon:** scientificName: Ametadoria mauriciogurdiani; phylum: Arthropoda; class: Insecta; order: Diptera; family: Tachinidae; genus: Ametadoria; specificEpithet: mauriciogurdiani; scientificNameAuthorship: Fleming & Wood, 2015; **Location:** continent: Central America; country: Costa Rica; countryCode: CR; stateProvince: Guanacaste; county: Area de Conservación Guanacaste; locality: Sector Pitilla; verbatimLocality: Casa Roberto; verbatimElevation: 520; verbatimLatitude: 11.011; verbatimLongitude: -85.421; verbatimCoordinateSystem: Decimal; decimalLatitude: 11.011; decimalLongitude: -85.421; **Identification:** identifiedBy: Fleming & Wood; dateIdentified: 2014; **Event:** samplingProtocol: reared from caterpillar of unidentified Zygaenidae; verbatimEventDate: 22-Oct-2011; **Record Level:** language: en; institutionCode: CNC; collectionCode: Insects; basisOfRecord: Pinned Specimen
- **Type status:** Paratype. **Occurrence:** occurrenceDetails: http://janzen.sas.upenn.edu; catalogNumber: DHJPAR0049522; recordedBy: D.H. Janzen, W. Hallwachs & Cirilo Umana; individualID: DHJPAR0049522; individualCount: 1; sex: F; lifeStage: adult; preparations: pinned; otherCatalogNumbers: 12-SRNP-76107; **Taxon:** scientificName: Ametadoria mauriciogurdiani; phylum: Arthropoda; class: Insecta; order: Diptera; family: Tachinidae; genus: Ametadoria; specificEpithet: mauriciogurdiani; scientificNameAuthorship: Fleming & Wood, 2015; **Location:** continent: Central America; country: Costa Rica; countryCode: CR; stateProvince: Guanacaste; county: Area de Conservación Guanacaste; locality: Sector Rincon Rain Forest; verbatimLocality: Finca Esmeralda; verbatimCoordinateSystem: Decimal; **Identification:** identifiedBy: Fleming & Wood; dateIdentified: 2014; **Event:** samplingProtocol: reared from caterpillar of unidentified Zygaenidae; **Record Level:** language: en; institutionCode: CNC; collectionCode: Insects; basisOfRecord: Pinned Specimen
- **Type status:** Paratype. **Occurrence:** occurrenceDetails: http://janzen.sas.upenn.edu; catalogNumber: DHJPAR0045546; recordedBy: D.H. Janzen, W. Hallwachs & Roster Moraga; individualID: DHJPAR0045546; individualCount: 1; sex: M; lifeStage: adult; preparations: pinned; otherCatalogNumbers: 11-SRNP-22559; **Taxon:** scientificName: Ametadoria mauriciogurdiani; phylum: Arthropoda; class: Insecta; order: Diptera; family: Tachinidae; genus: Ametadoria; specificEpithet: mauriciogurdiani; scientificNameAuthorship: Fleming & Wood, 2015; **Location:** continent: Central America; country: Costa Rica; countryCode: CR; stateProvince: Guanacaste; county: Area de Conservación Guanacaste; locality: Sector El Hacha; verbatimLocality: Sendero Congos; verbatimElevation: 290; verbatimLatitude: 11.021; verbatimLongitude: -85.525; verbatimCoordinateSystem: Decimal; decimalLatitude: 11.021; decimalLongitude: -85.525; **Identification:** identifiedBy: Fleming & Wood; dateIdentified: 2014; **Event:** samplingProtocol: reared from caterpillar of unidentified Zygaenidae; verbatimEventDate: 25-Oct-2011; **Record Level:** language: en; institutionCode: CNC; collectionCode: Insects; basisOfRecord: Pinned Specimen
- **Type status:** Paratype. **Occurrence:** occurrenceDetails: http://janzen.sas.upenn.edu; catalogNumber: DHJPAR0046414; recordedBy: D.H. Janzen, W. Hallwachs & Elda Araya; individualID: DHJPAR0046414; individualCount: 1; sex: M; lifeStage: adult; preparations: pinned; otherCatalogNumbers: 11-SRNP-3905; **Taxon:** scientificName: Ametadoria mauriciogurdiani; phylum: Arthropoda; class: Insecta; order: Diptera; family: Tachinidae; genus: Ametadoria; specificEpithet: mauriciogurdiani; scientificNameAuthorship: Fleming & Wood, 2015; **Location:** continent: Central America; country: Costa Rica; countryCode: CR; stateProvince: Guanacaste; county: Area de Conservación Guanacaste; locality: Sector San Cristobal; verbatimLocality: Puente Palma; verbatimElevation: 460; verbatimLatitude: 10.9163; verbatimLongitude: -85.37869; verbatimCoordinateSystem: Decimal; decimalLatitude: 10.9163; decimalLongitude: -85.379; **Identification:** identifiedBy: Fleming & Wood; dateIdentified: 2014; **Event:** samplingProtocol: reared from caterpillar of unidentified Zygaenidae; verbatimEventDate: 11/Sep/2011; **Record Level:** language: en; institutionCode: CNC; collectionCode: Insects; basisOfRecord: Pinned Specimen

#### Description

**Male** (Fig. 4a, b, c). **Head** (Fig. 4c): frontal vitta dark black, narrowed apically to slightly less than the width of the ocellar triangle, fronto-orbital plate as wide as frontal vitta; ocellar bristles well developed, proclinate, half as long as arista; frontal bristles extending below level of arista by width of two bristles; small black setulae intermingled with frontal bristles all along the parafrontal, extending to level of upper margin of pedicel; antenna black; arista black and bare, minutely pubescent at its base and bare at its tip; proclinate orbital bristles absent; parafrontal mostly silver except for gold tinge along the margin of the frontal vitta, surrounding frontal bristles; parafacial silver; palpus yellow and haired at its tip; gena 1/10 height of head. **Thorax** (Fig. 4a, b): grayish-gold when viewed dorsally, with four longitudinal dark gray vittae; scutellum bearing silver tomentosity over its entirety, this tomentosity shifting in color to velvety black when viewed dorsally (extending to underside of scutellum). Legs black; anteroventral surface of hind femur bearing 5–6 long erect bristles of irregular length and irregularly spaced, anterodorsal surface of hind tibia with regularly spaced comb of bristles 3X as long as tibia is wide; anteroventral surface of hind tibia lacking tightly adpressed, short, regularly spaced hairs but with 5–6 long bristles of irregular length and erratic spacing. Wing (Fig. 4a): pale smoky yellowish in color; calypters dark amber yellow translucent, haired along their margins. **Abdomen** (Fig. 4a): ground color of abdomen dark brown, almost black, dorsally, appearing dark velvety black medially, with bright silver, gold tinged tomentose bands covering up to 1/3 of tg 3, 4, and 5, these bands changing to silver and wrapping around to the underside of the abdomen; bands appearing broken dorsocentrally; only tergites 3, 4 and 5 possess median marginal bristles. Ventrolateral surface concolorous; sex patches present on ventral surface of tg 4 and tg 5. **Terminalia** (Figs 5c, 6c, 7c): sternite 5 with deep wide U-shaped median cleft, forming two rounded outer lobes, these covered in dense tomentum; lobes of sternite 5 bearing 5 long bristles marginally, the longest approximately 2X as long as other bristles present on sternite 5. Cerci almost flat when viewed laterally, but with a distinct angular bend approximately halfway, creating a very slight forward angle on the anterior half; cerci narrow and finger-like in dorsal view, not fused medially, maintaining an almost equidistant separation along their entire length; cerci covered in long setulae dorsally, tapering at around 4/5 of their length, with only the apex bare. Surstylus short and truncate, approximately 2/3 as long as cerci; surstylus rounded and sub-triangular with a slight hook to the tip when viewed laterally; lateral surface of surstylus slightly haired, inferior margin apically with 3–5 stout bristles, confined to anterior apex, these bristles being much stouter and thicker than any others present on the genitalia. Epandrium, when viewed laterally, not extending backwards, 2/3 length of cerci. Phallus short and stout, approximately same length as cerci. Postgonite 1/3 as long as phalloapodeme and with a strong forward curve.

**Female** (Fig. 4d, e, f). **Head** (Fig. 4f): frontal vitta dark black, narrowed apically to less than the width of the ocellar triangle, fronto-orbital plate as wide as frontal vitta; frontal bristles not reaching below level of pedicel; 2 proclinate orbital bristles present; parafrontal with gold over more than half its surface, extending down to lower proclinate orbital bristle, gold color then tapering to just along facial margin down to the lowest frontal bristle, sparsely populated with minute setulae over its entire surface; parafacial silver; palpi orange and haired. **Thorax** (Fig. 4d, e): golden when viewed dorsally with four longitudinal gray vittae, these only slightly visible post-suturally, appearing broken at thoracic suture; four post-sutural dorsocentral bristles; scutellum bearing gold tomentosity distally, and black proximal to the mesothorax. Legs and wings as in males. **Abdomen** (Fig. 4d​): abdominal tergites dark velvety black medially, with a bright brassy band covering 2/3 or more of tergal surface, these bands changing to silver as they wrap around to the underside of the abdomen. Tergites 3, 4 and 5 possessing median marginal bristles, syntergite 1+2 without such bristles.

#### Diagnosis

Fronto-orbital plate with gold tomentum, prominent along margin of the frontal vitta and surrounding the frontal bristles; parafacial silver; calypters entirely dark amber yellow translucent; anterodorsal surface of hind tibia with a regularly spaced comb of bristles 3X as long as tibia is wide; abdominal tergites with black ground color bearing bright silver tomentose bands covering up to 1/3 of tergal surface; syntergite 1+2 lacking median marginal bristles; ventrolateral surface of syntergite 1+2, tg 3, and tg 4 concolorous with black ground color of dorsal surface. Epandrium, when viewed laterally, not extending backwards, 2/3 length of cerci. Phallus short and stout, approximately same length as cerci. Postgonite 1/3 as long as phalloapodeme with a strong forward curve. *Ametadoria mauriciogurdiani* is distinguished from *A. fuliginipennis*, which is also black in overall ground color, by the following traits: tergal tomentosity in *A. fuliginipennis* is reduced on tg 3 relative to *A. mauriciogurdiani* and increased to over half of tergal surface on tg 4 and tg 5; abdominal tomentosity in *A. fuliginipennis* is of a bright golden color in comparison with the more silver tone in *A. mauriciogurdiani*.

#### Etymology

*Ametadoria mauriciogurdiani* is named in recognition of Mauricio Gurdián Chamorro for his contributions to the accounting team for Area de Conservación Guanacaste, the forest this fly lives in.

#### Distribution

Costa Rica, ACG, Prov. Guanacaste and Alajuela, rain forest and dry forest, 123–675 m elevation.

#### Ecology

Host: *Ametadoria mauriciogurdiani* was reared from at least six species of unidentified Zygaenidae feeding on Dilleniaceae and Marcgraviaceae.

## Discussion

### Barcoding Results

The DNA barcode sequences recovered from ACG *Ametadoria* species display the characteristic strong AT bias of insect mitochondrial DNA (mean percent GC content 30.21, SE 0.06) and no insertions or deletions. Within-species variation was low (mean distance of 0.06%) compared to between-species variation (mean distance 7.26%). All values for DNA barcode variation were calculated within BOLD and can be re-calculated in the future as more specimens are recovered from the ACG inventory and added to the DNA library.

## Supplementary Material

XML Treatment for Ametadoria karolramosae

XML Treatment for Ametadoria leticiamartinezae

XML Treatment for Ametadoria mauriciogurdiani

## Figures and Tables

**Figure 2a.**
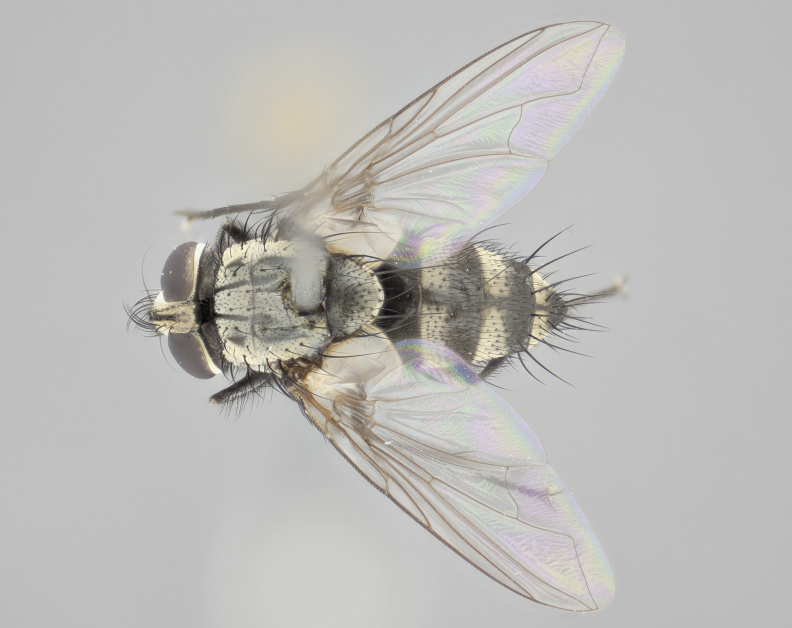
Habitus, dorsal

**Figure 2b.**
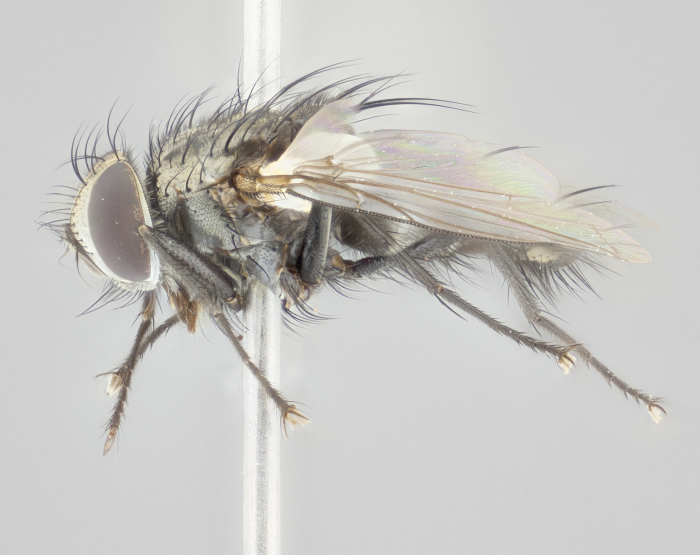
Habitus, lateral

**Figure 2c.**
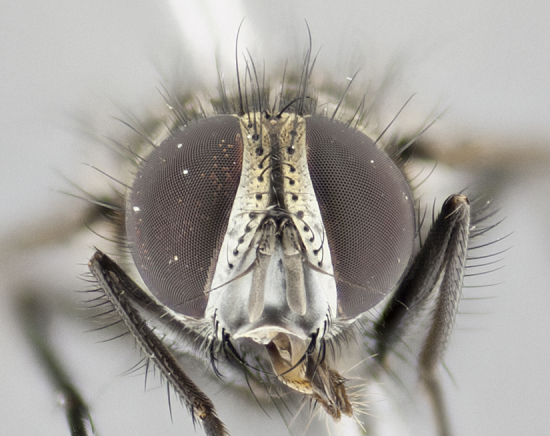
Head, frontal

**Figure 2d.**
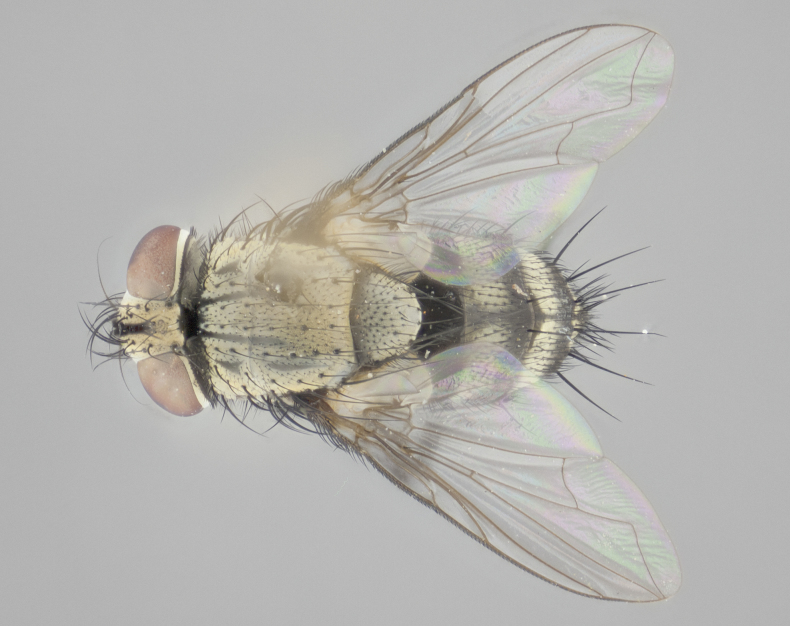
Habitus, dorsal

**Figure 2e.**
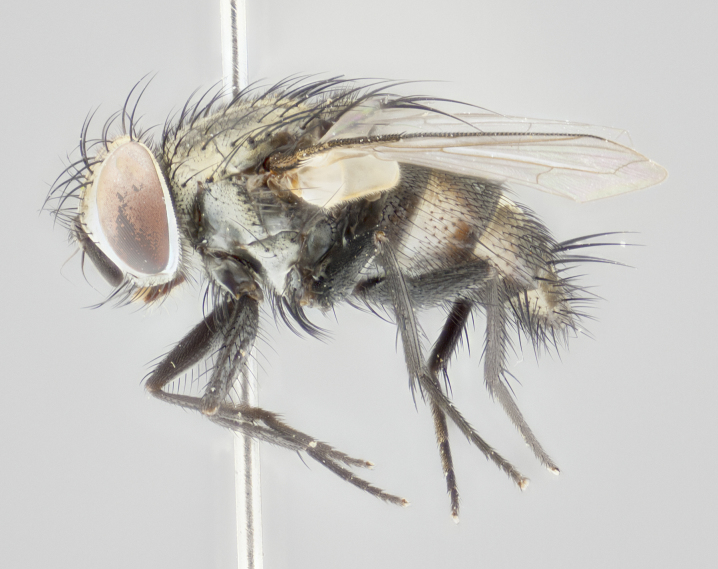
Habitus, lateral

**Figure 2f.**
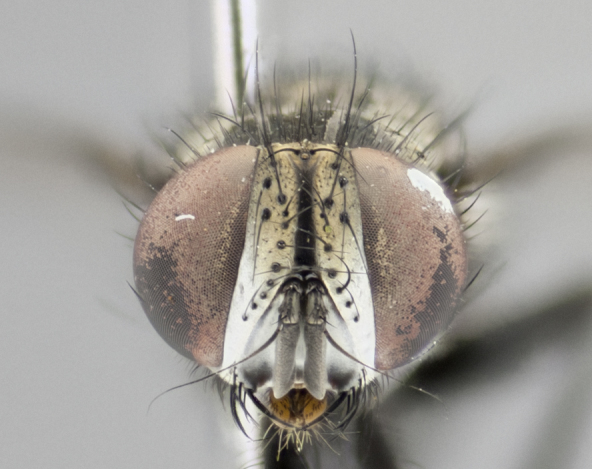
Head, frontal

**Figure 3a.**
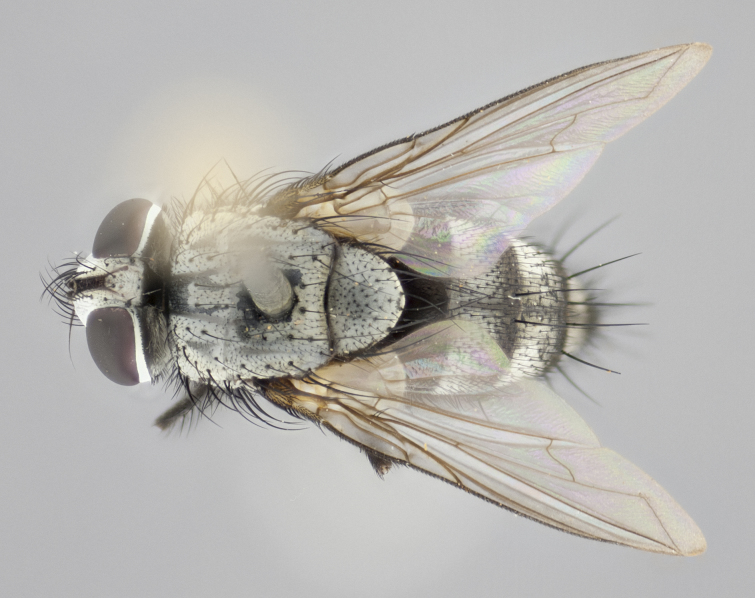
Habitus, dorsal

**Figure 3b.**
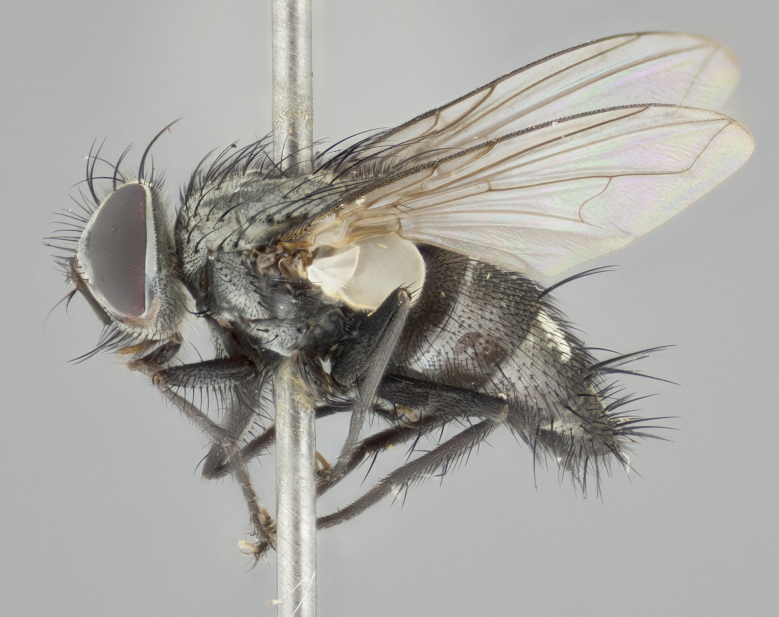
Habitus, lateral

**Figure 3c.**
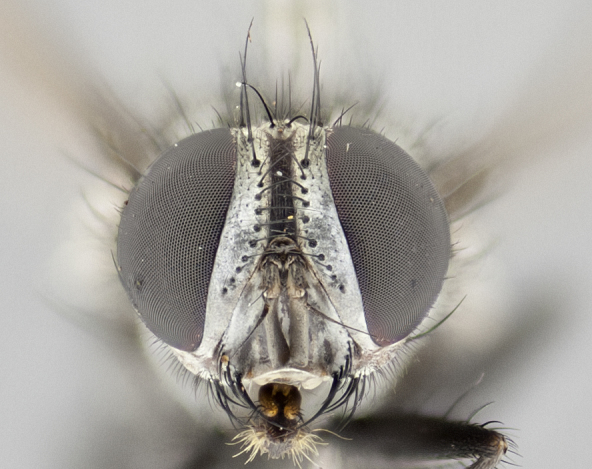
Head, frontal

**Figure 3d.**
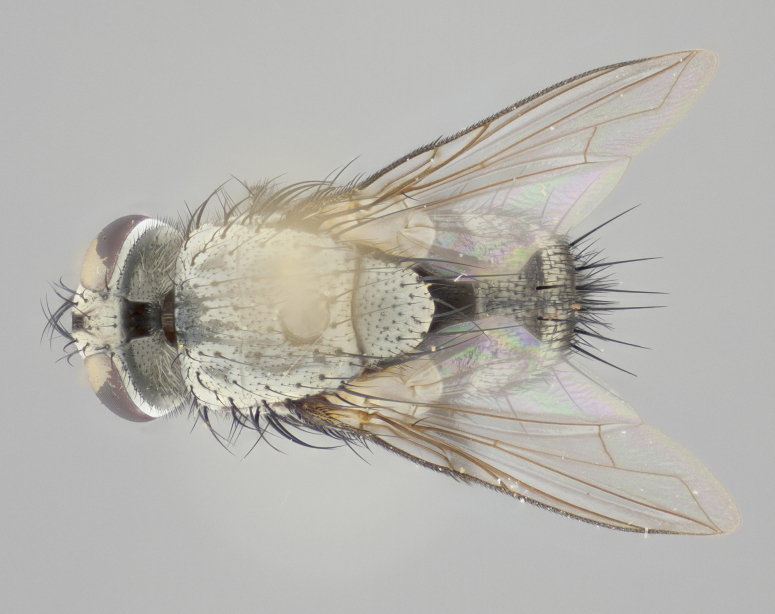
Habitus, dorsal

**Figure 3e.**
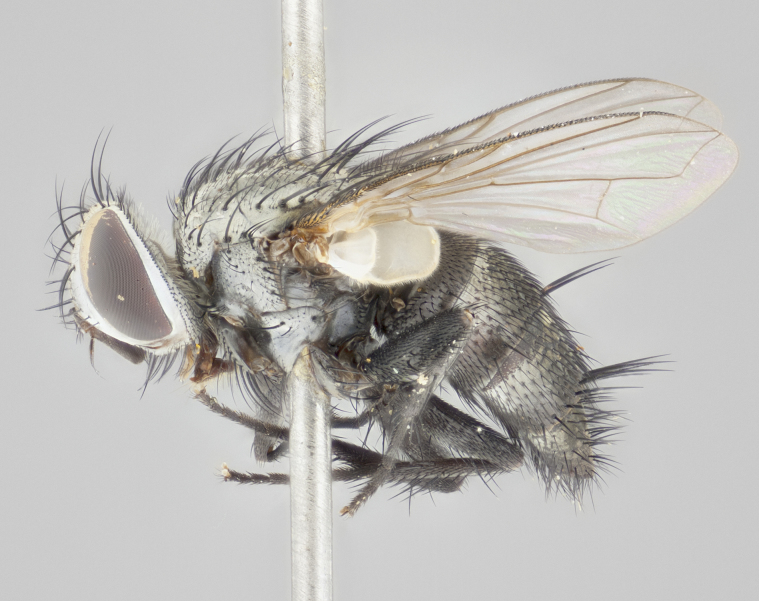
Habitus, lateral

**Figure 3f.**
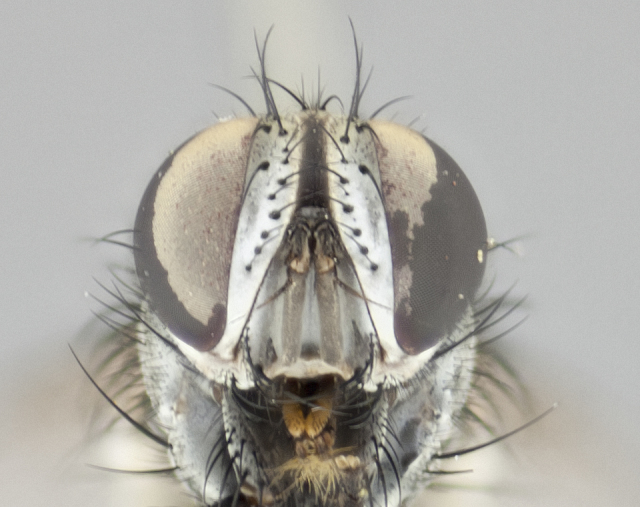
Head, frontal

**Figure 4a.**
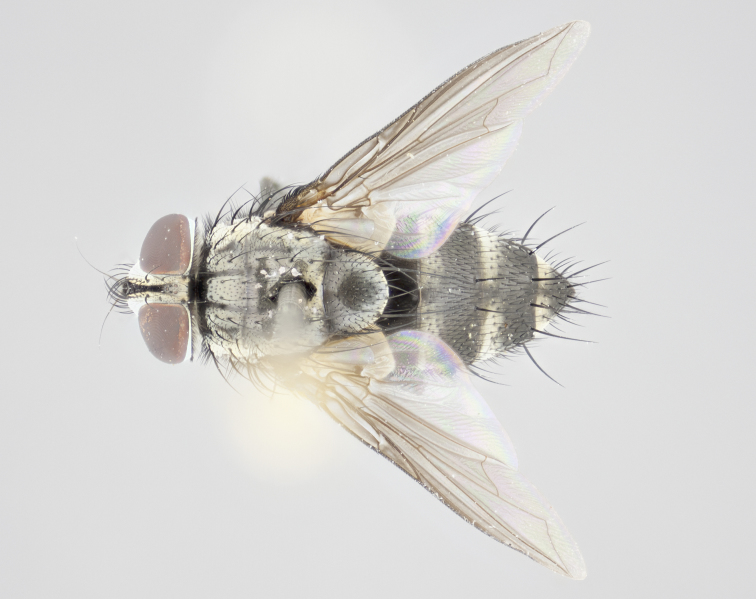
Habitus, dorsal

**Figure 4b.**
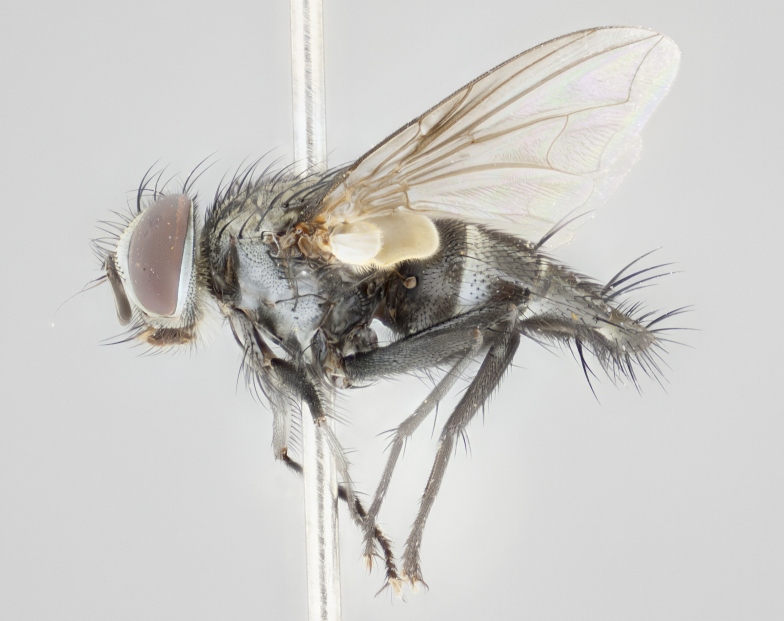
Habitus, lateral

**Figure 4c.**
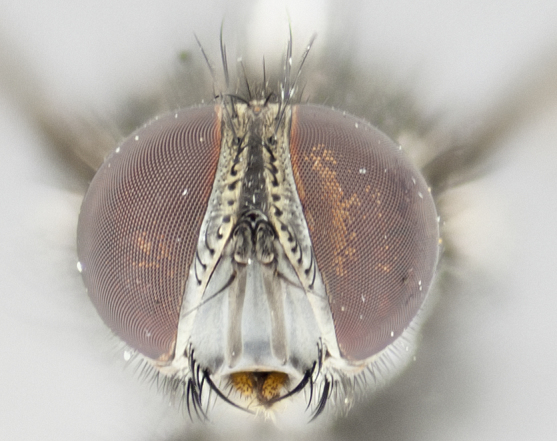
Head, frontal

**Figure 4d.**
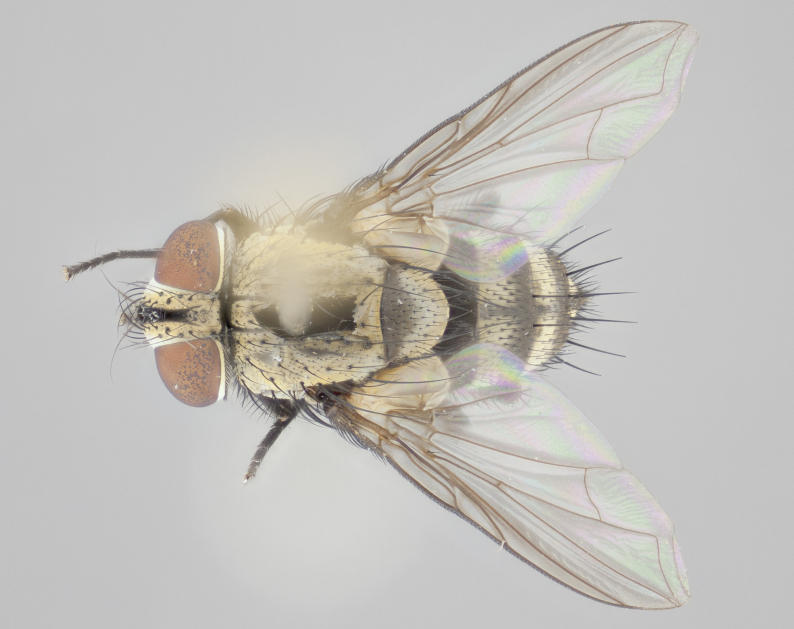
Habitus, dorsal

**Figure 4e.**
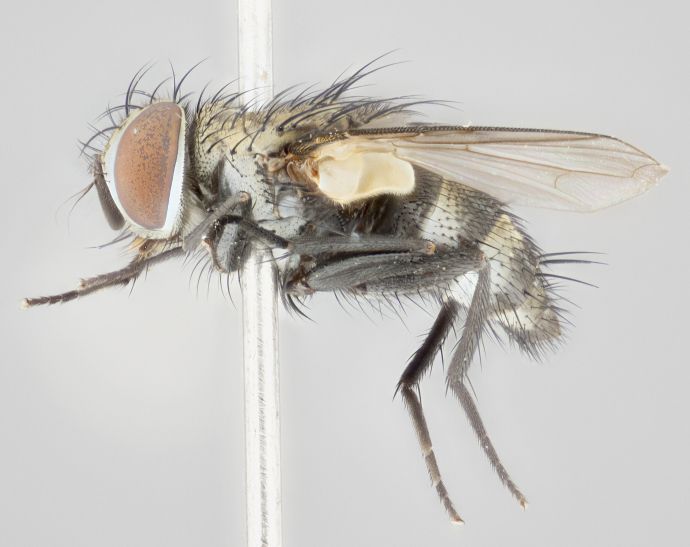
Habitus, lateral

**Figure 4f.**
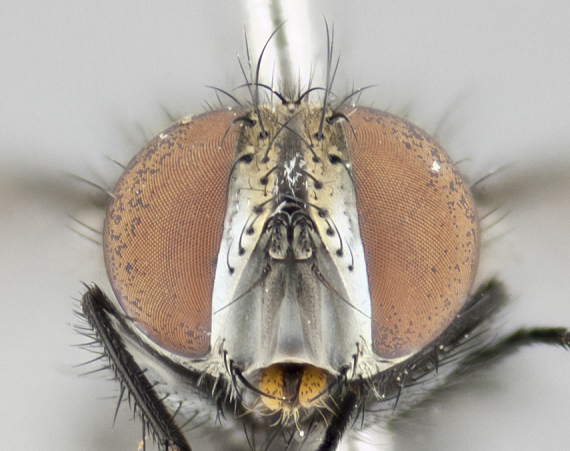
Head, frontal

**Figure 5a.**
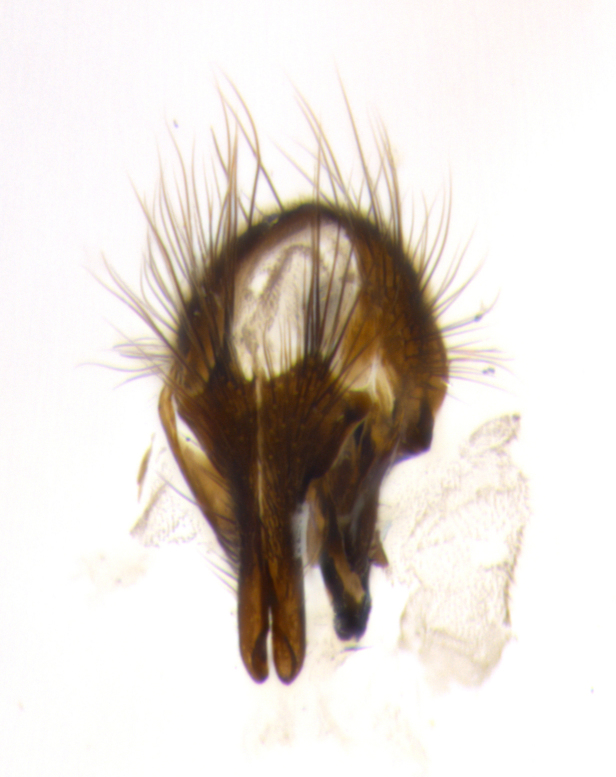
*Ametadoria karolramosae* (paratype, DHJPAR0040114)

**Figure 5b.**
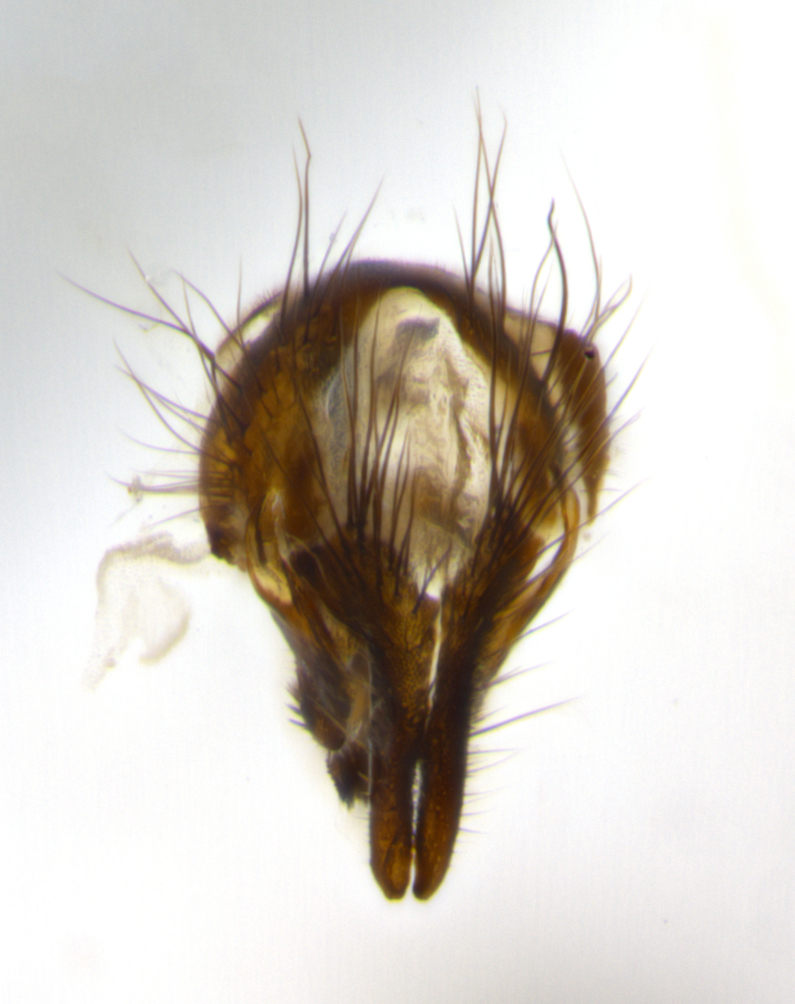
*Ametadoria leticiamartinezae* (paratype, DHJPAR0018978)

**Figure 5c.**
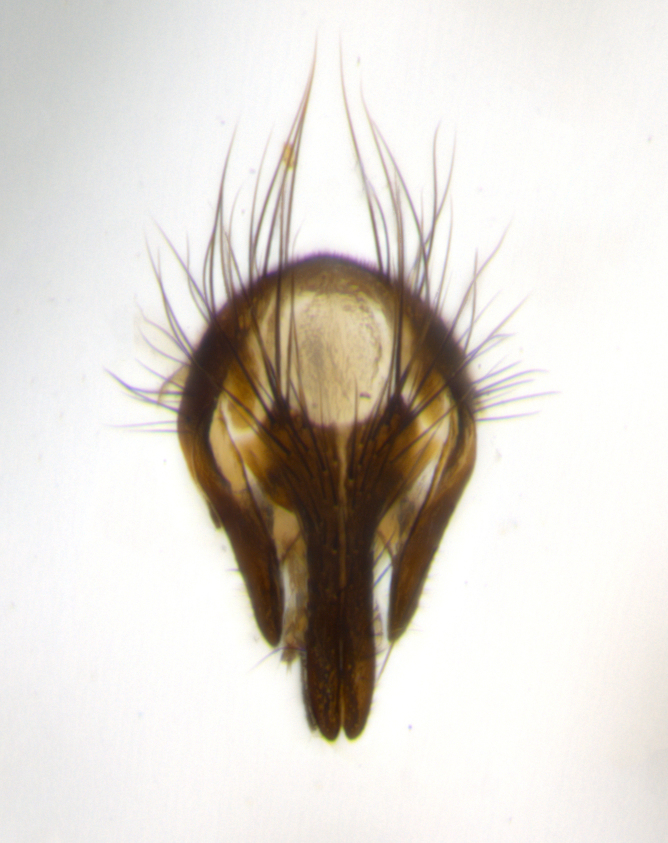
*Ametadoria mauriciogurdiani* (paratype, DHJPAR0049512)

**Figure 6a.**
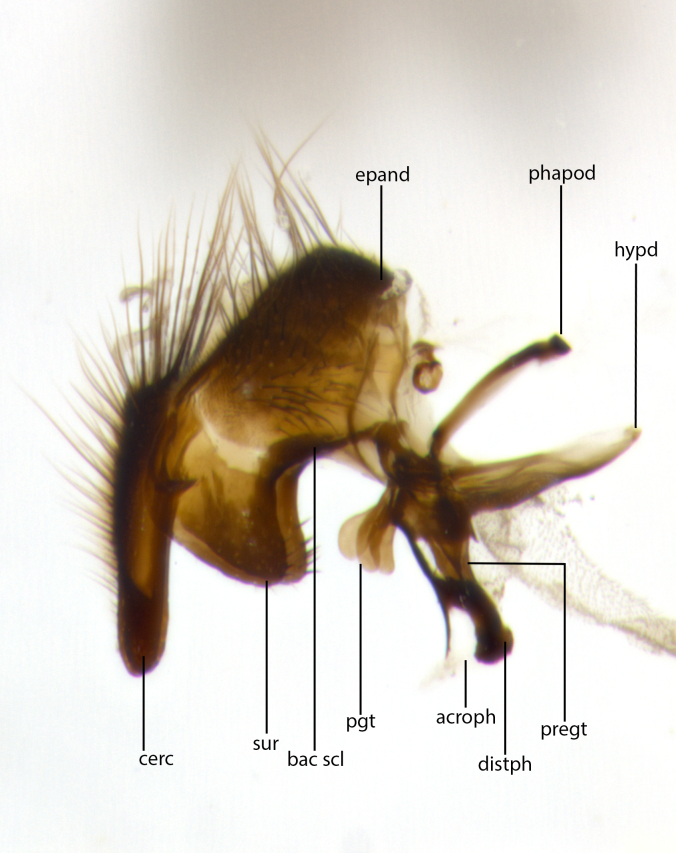
*Ametadoria karolramosae* (paratype, DHJPAR0040114)

**Figure 6b.**
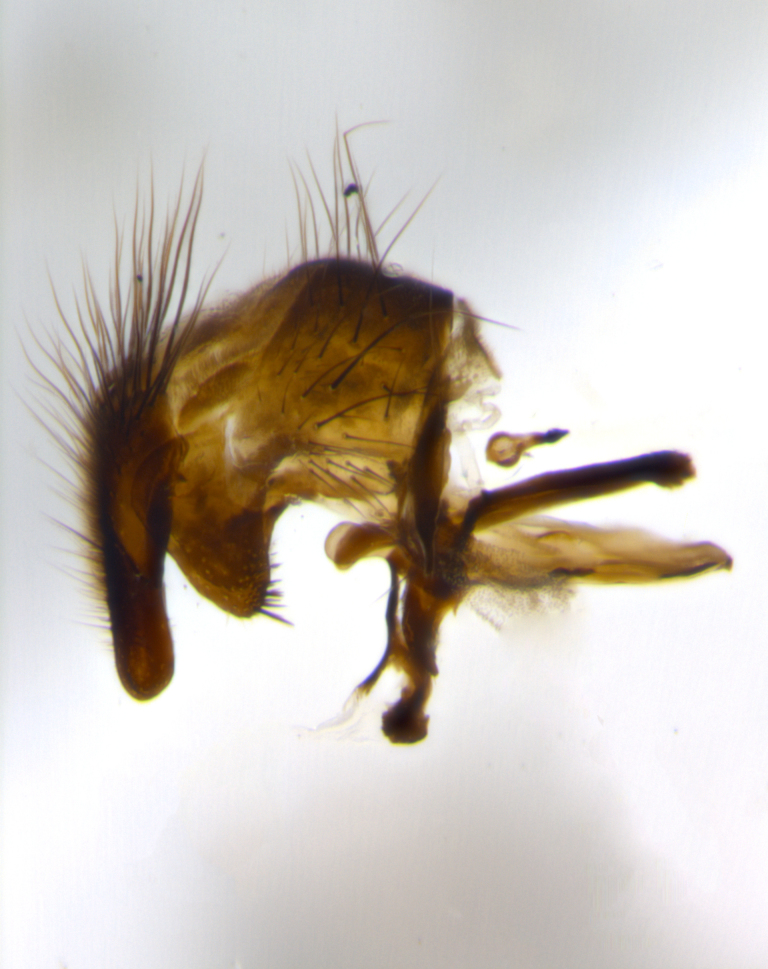
*Ametadoria leticiamartinezae* (paratype, DHJPAR0018978)

**Figure 6c.**
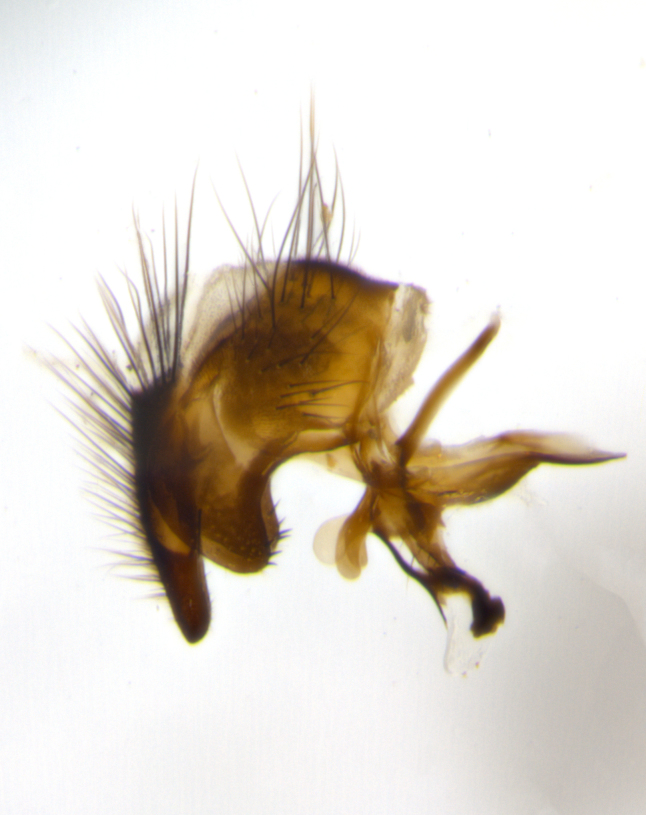
*Ametadoria mauriciogurdiani* (paratype, DHJPAR0049512)

**Figure 7a.**
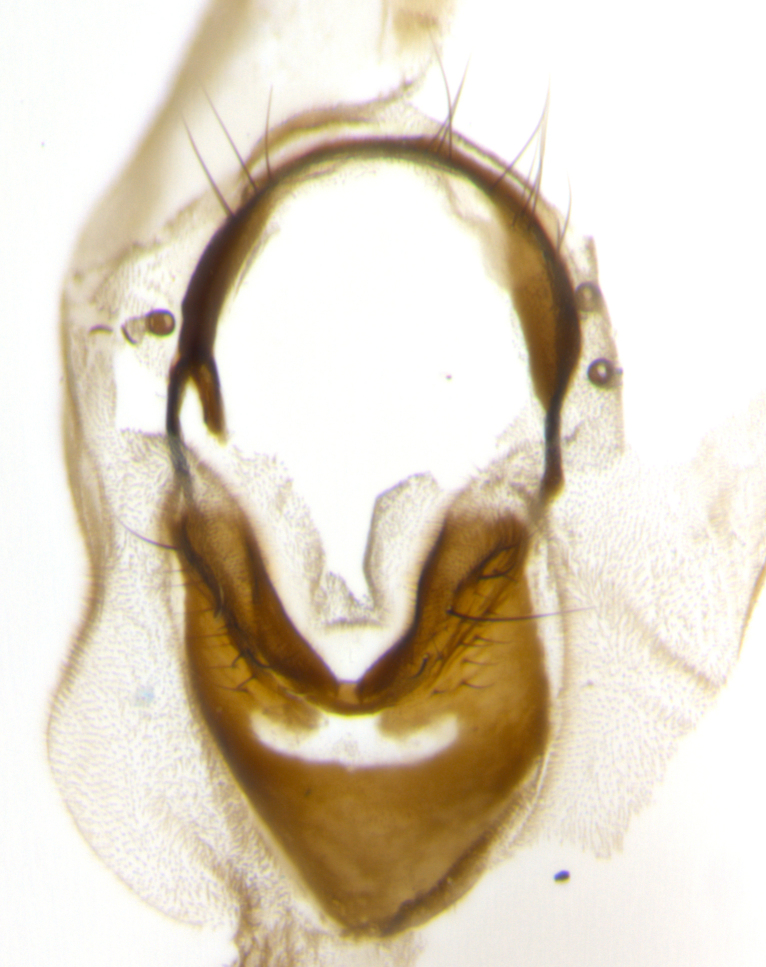
*Ametadoria karolramosae* (paratype, DHJPAR0040114)

**Figure 7b.**
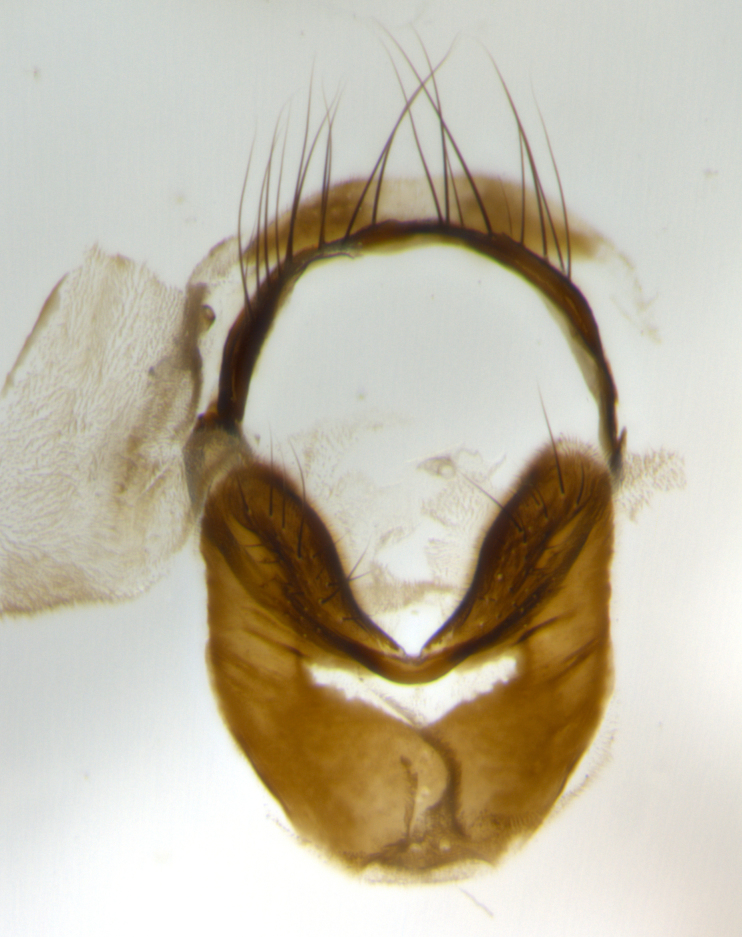
*Ametadoria leticiamartinezae* (paratype, DHJPAR0018978)

**Figure 7c.**
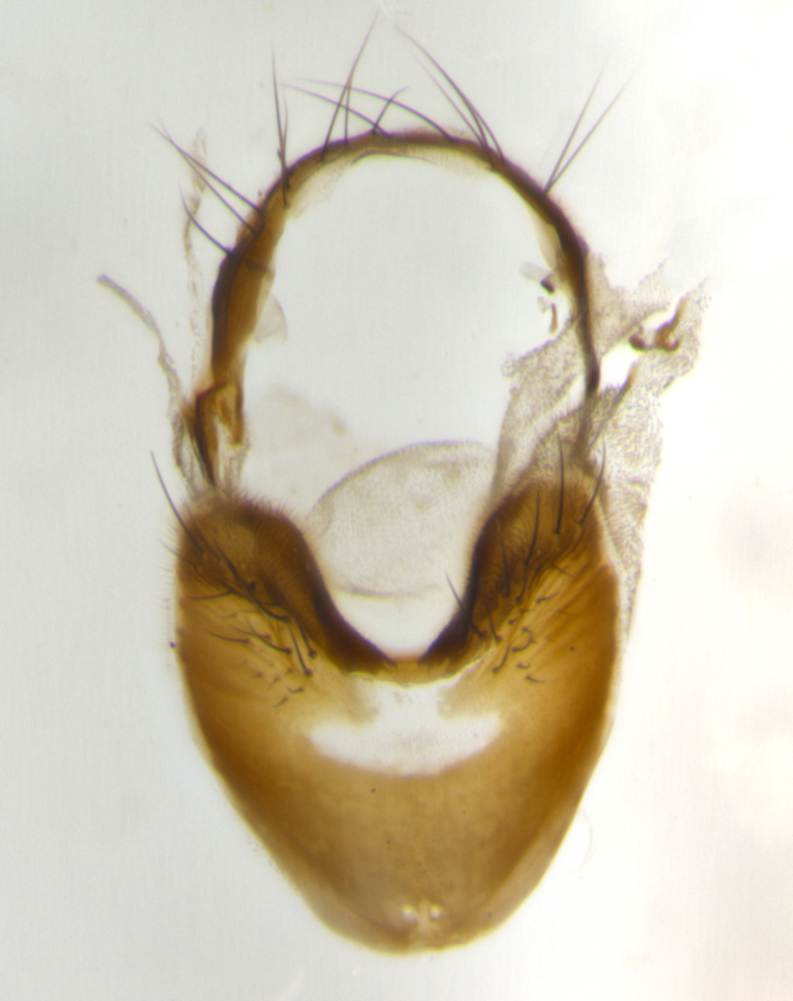
*Ametadoria mauriciogurdiani* (paratype, DHJPAR0049512)
